# A spatial adaptive multi-scale ConvNeXt framework for robust, calibrated, and statistically validated multi-class retinal OCT classification

**DOI:** 10.3389/frai.2026.1898978

**Published:** 2026-08-31

**Authors:** Mithun Vijayan, Goutham Veerapu, Nisha J. S.

**Affiliations:** School of Electronics Engineering, Vellore Institute of Technology, Vellore, India

**Keywords:** ConvNeXt, deep learning, interpretability, model calibration, multi-scale feature extraction, optical coherence tomography (OCT), retinal disease classification, robustness analysis

## Abstract

Accurate classification of retinal diseases from optical coherence tomography (OCT) images is important for clinical decision support. However, many studies in deep learning put the primary focus on enhancing accuracy and often provide limited attention to calibration and robustness under real-world variations. This work presents an enhanced ConvNeXt-Tiny framework incorporating a Spatial Adaptive Multi-scale Convolution (SAMC) module for retinal OCT classification. Rather than introducing a new convolutional operation, SAMC combines established multi-scale dilated convolution and feature fusion principles in a lightweight stage-wise configuration within the ConvNeXt hierarchy to refine representations of retinal abnormalities occurring at different spatial scales. The model is evaluated on the OCT-C8 dataset with eight retinal disease classes. It obtains a test accuracy of 98.39% and a macro F1 score of 0.9839. Reliability of classification was also analyzed by measuring Expected Calibration Error (ECE) and Brier score. Temperature scaling is applied as a *post-hoc* calibration method, while Monte Carlo dropout is used to estimate predictive variability under stochastic inference. Robustness is evaluated under noise, blur, and illumination changes, where minimal performance drop was observed. Computational complexity analysis is performed in terms of model parameters, floating-point operations, and inference efficiency to assess the practicality of the proposed framework. Statistical significance is confirmed using McNemar's test. Interpretability analysis with Grad-CAM and LIME reveals the model attends to clinically significant regions of the retina. Overall, the proposed ConvNeXt-SAMC framework achieves accurate retinal disease classification, while additional calibration, uncertainty, robustness, statistical, and interpretability analyses provide a broader assessment of model behavior.

## 1 Introduction

**Figure 1:**
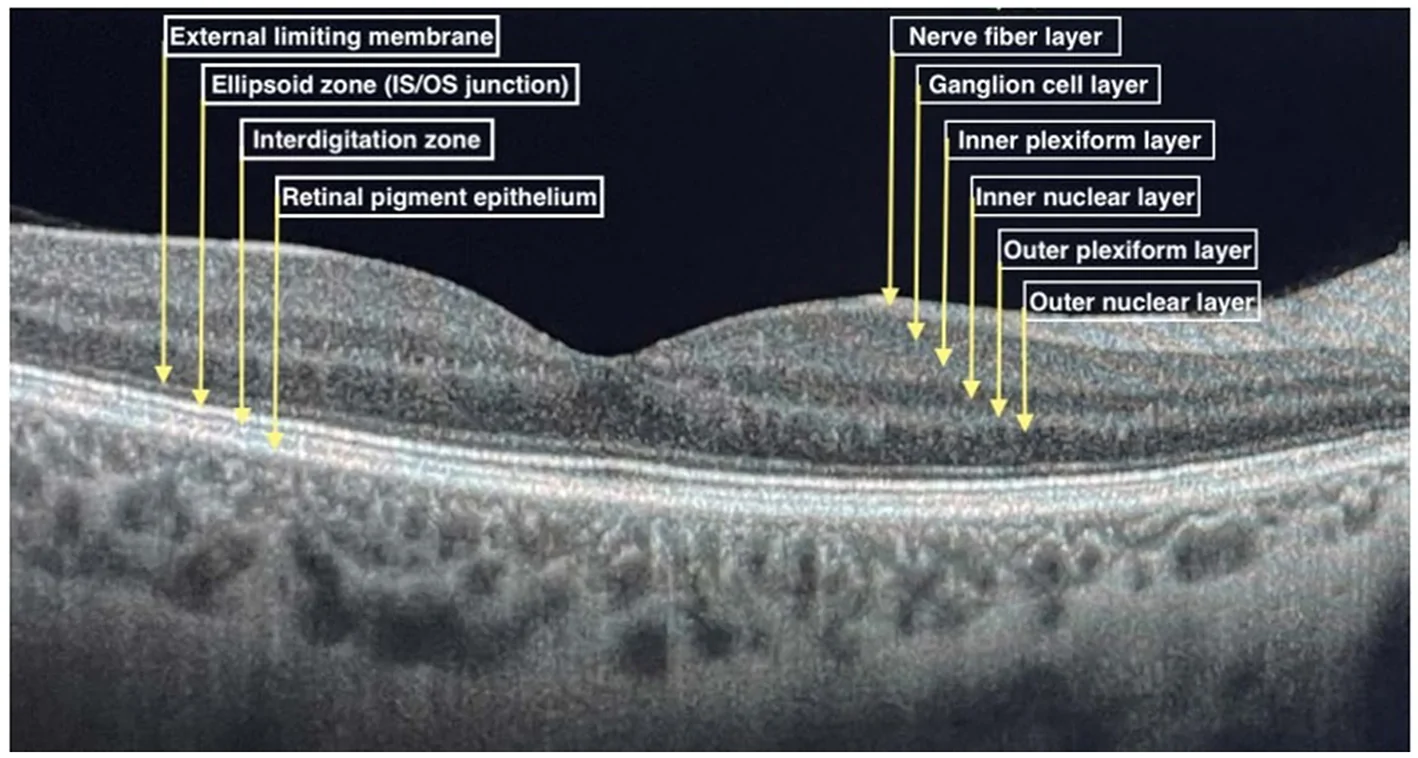
OCT image of the central retina from a normal subject. Key retinal layers and structures are highlighted with arrows for reference. Reproduced from (Mimier-Janczak et al. 2023) under the CC BY-NC-SA 4.0 license.

Retinal diseases are among the leading causes of irreversible visual impairment worldwide (World Health Organization, 2024; Steinmetz et al., 2021). Conditions such as age-related macular degeneration (AMD), diabetic retinopathy (DR), choroidal neovascularization (CNV), drusen, central serous retinopathy (CSR), diabetic macular edema (DME), macular hole (MH), and normal retinal variations exhibit subtle structural differences that can be effectively visualized using OCT (Drexler and Fujimoto, 2008; Huang et al., 1991). OCT generates detailed cross-sectional views of retinal anatomy which helps clinicians to detect microstructural abnormalities at early stages. But manual interpretation of OCT scans is prone to inter-observer variability (De Fauw et al., 2018). Automated deep-learning-based classification systems can assist clinicians by providing quick and reliable diagnostic support. Retinal OCT images consist of multiple anatomically distinct layers, each associated with specific functional and pathological roles. Structural changes including fluid buildup, layer wise deformation, and tissue disruption can happen at different depths and in different areas. This necessitates effective multi scale feature representation for reliable automated analysis. Figure 1 illustrates the layered retinal structure in a normal OCT scan.

In recent years, convolutional neural networks (CNNs) and transformer-based architectures have achieved significant progress in retinal OCT classification (Kermany et al., 2018; De Fauw et al., 2018). Architectures such as ResNet (He et al., 2016), EfficientNet (Tan and Le, 2019), and DenseNet (Huang et al., 2017) have demonstrated strong discriminative capabilities. ConvNeXt has emerged as a modernized convolutional architecture recently. It combines the design concepts inspired by vision transformers with convolutional efficiency (Liu et al., 2022). Lightweight models have also been proposed to improve computational efficiency while maintaining high accuracy. Less attention has been given to multi-scale feature representation, robustness under image perturbations, prediction calibration, and statistical validation of improvements (Guo et al., 2017; Ovadia et al., 2019). The main drawbacks of the current OCT classification techniques are outlined in Table 1, along with how the suggested framework addresses these challenges.

**Table 1:** Comparative analysis of OCT classification methods and the proposed ConvNeXt–SAMC framework.

Category	Representative studies	Limitations of existing methods	Proposed solution
Conventional CNN-based OCT classification	Kermany et al.; ResNet (He et al., 2016); DenseNet (Huang et al., 2017); EfficientNet (Tan and Le, 2019)-based	Operate with fixed receptive fields, which limits the ability to capture retinal abnormalities that vary significantly in size and spatial distribution.	Introduces stage-wise SAMC refinement to capture fine and broad retinal structures.
Attention-based OCT classification models	SE-Net (Hu et al., 2018; Hayat, 2024), CBAM (Zhang et al., 2023), and other attention-augmented CNNs	Primarily focus on channel or spatial weighting without explicitly enhancing multi-scale spatial feature extraction; often increase complexity.	Uses lightweight multiscale spatial convolution in backbone stages to improve spatial representation without introducing heavy attention overhead.
Multi-branch and feature pyramid fusion models	Multi-path CNN and feature pyramid network (Yang et al., 2023; Rasti et al., 2017; Das et al., 2019)-based	Use static feature fusion strategies that do not dynamically refine hierarchical feature representations during progressive encoding.	Introduces stage-wise adaptive multi-scale refinement.
Transformer and hybrid CNN-Transformer	Vision Transformer (Liu et al., 2023), Swin Transformer (Liu et al., 2021) based	Require large computational resources and higher parameter counts, limiting suitability for real time or resource constrained deployment.	Enhances contextual modeling while preserving ConvNeXt efficiency.
Existing OCT studies focused primarily on accuracy	Majority of recent deep learning-based research	Lack comprehensive evaluation of model reliability, calibration, robustness, and statistical significance.	Includes calibration assessment, uncertainty estimation using MC Dropout, robustness evaluation, and statistical validation using McNemar's test.
Limited interpretability validation	Existing explainable AI-based OCT studies	Apply explainability methods only for visualization without integrating interpretability into model validation.	Provides systematic interpretability analysis using Grad-CAM (Selvaraju et al., 2017) and LIME (Ribeiro et al., 2016) to verify that the model focuses on clinically meaningful retinal regions.

**Figure 2:**
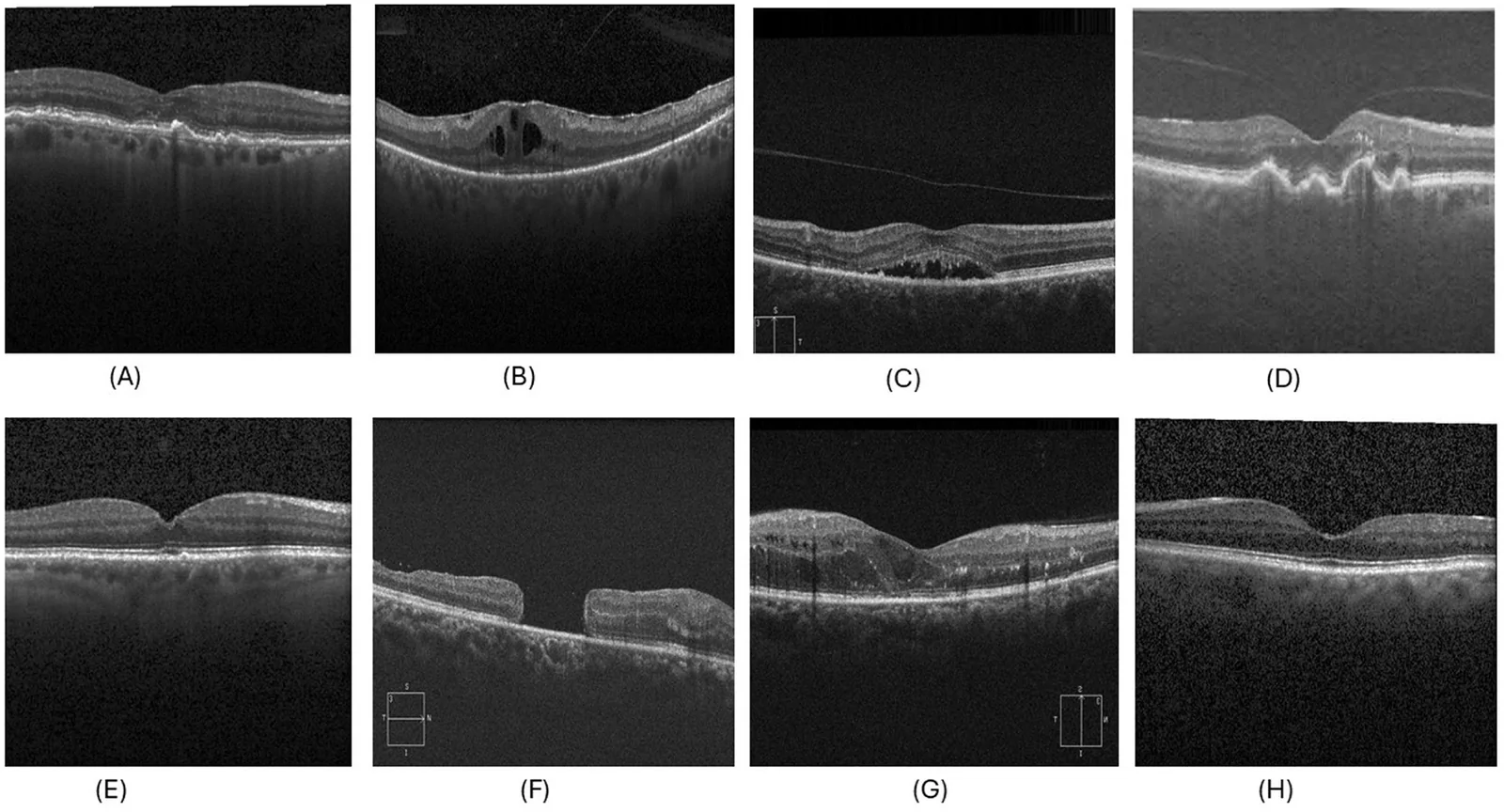
Representative B-scan images from the OCT C8 dataset showing variability in retinal layer organization, including disruptions in continuity and the presence of localized and diffuse abnormalities of different spatial extents. **(A)** CNV. **(B)** DME. **(C)** CSR. **(D)** AMD. **(E)** NORMAL. **(F)** MH. **(G)** DR. **(H)** DRUSEN.

Abnormalities in retinal OCT are observed at various spatial scales. It spans from localized deposits to more extensive structural disruptions. Figure 2 presents representative B-scan images showing these variations. Standard convolutional architectures often rely on fixed receptive fields. This limits their ability to capture such heterogeneous structures effectively. In addition, reliable clinical deployment requires robustness to image perturbations and calibrated confidence estimates for safer decision support (Guo et al., 2017).

To address these challenges, the ConvNeXt-Tiny backbone is extended with a Spatial Adaptive Multi-scale Convolution (SAMC) module. The contribution of this design lies in the lightweight, stage-wise integration of multi-scale spatial refinement within the ConvNeXt hierarchy, rather than in the individual use of dilated convolution or feature fusion. Parallel depthwise convolutions with different dilation rates capture complementary spatial contexts, while feature fusion is applied to refine the resulting representations before subsequent hierarchical processing.

In addition to the architectural changes, the model is evaluated under different settings. Accuracy and macro F1 score are reported, along with robustness to noise, blur, and brightness changes. The reliability is evaluated using ECE, Brier Score, and temperature scaling. The uncertainty is computed using Monte Carlo dropout method. For comparing the proposed system with the baseline system, the McNemar's test is used. Grad CAM (Selvaraju et al., 2017) and LIME (Ribeiro et al., 2016) are used to visualize the regions that influence the predictions.

The contributions of this study are as follows:

1. A lightweight stage-wise SAMC configuration is embedded within the ConvNeXt-Tiny backbone to progressively refine the representation of retinal abnormality at multiple spatial resolutions.
2. A comprehensive evaluation framework is used to assess prediction reliability, including calibration performance, uncertainty estimation, robustness under controlled perturbations, computational efficiency, and statistical significance beyond conventional classification metrics.
3. The statistical significance of the observed performance improvement is validated using McNemar's test by comparing the proposed ConvNeXt-SAMC model with the baseline ConvNeXt-Tiny model.
4. Model interpretability is investigated using Grad-CAM and LIME to verify the localization of clinically meaningful retinal features and improve the transparency of model predictions.

Experimental results show that the proposed ConvNeXt-SAMC model achieves good classification performance, and at the same time has reliable predictions and robust behavior under different conditions. These results show its potential use in real-world clinical decision support scenarios.

## 2 Related works

### 2.1 Retinal OCT classification using CNN

Deep learning for retinal OCT analysis has resulted in considerable advances in automated diagnosis performance over the last decade. Early CNN based studies showed that transfer learning from large scale natural image datasets can be effectively adapted to ophthalmic imaging tasks. A well known study by (Kermany et al. 2018) demonstrated multiclass retinal disease classification using InceptionV3, reporting performance comparable to expert ophthalmologists on the OCT2017 dataset.

Deeper convolutional backbones have been investigated as a way to improve representation learning. Residual networks (He et al., 2016) and densely connected models (Huang et al., 2017) improve gradient propagation and enable effective feature reuse. EfficientNet (Tan and Le, 2019) follows a different approach by scaling depth, width, and resolution together. Reported accuracies for these models often range from 97%–99% on curated OCT datasets. However, such performance is typically measured under controlled conditions with limited variability across imaging devices.

Although high accuracy is often reported, many of these models depend on conventional hierarchical feature extraction with fixed receptive fields. This makes it hard for the models to detect local anomalies as well as changes in context across retinal layers. Robustness to changes in acquisition conditions, as well as to real world disturbances, has not been much addressed before.

### 2.2 Contextual and multi-scale modeling strategies

Retinal images contain pathological features of varying sizes, from small focal lesions to more extensive structural abnormalities. This diversity presents a challenge for image analysis, as relevant information may be present at different levels of spatial details. This motivates the development of contextual and multi-scale modeling approaches.

Multi scale operations such as dilated convolutions and pyramid pooling are widely used in medical image analysis. Methods such as Atrous Spatial Pyramid Pooling (ASPP) (Chen et al., 2018) employ multiple dilation rates to capture contextual information. Also Feature Pyramid Networks (FPN) (Lin et al., 2017) integrate representations across feature levels to improve contextual understanding.

In OCT analysis, multi branch CNN architectures and feature fusion networks improve discrimination of subtle pathological patterns (Rasti et al., 2017; Das et al., 2019). Recently developed multi-scale architectures like WaveNet-SF (Cheng et al., 2025) make use of wavelet feature decomposition that allows for frequency aware representation extraction. But, MSLI-Net (Qi et al., 2025) combines multi-level together with multi-scale feature fusion to enable better contextual learning for retinal OCT classification. Many methods depend on using static feature fusion or parallel branches. Many methods depend on static feature fusion or parallel branches. This increases complexity while limiting progressive refinement across hierarchical stages.

These studies show the proven effectiveness of dilated convolution, pyramid representations and multi-level feature fusion. The proposed work is based on these well-established techniques, without changing their fundamental formulations. Instead, it propose a stage-wise and lightweight integration of multi-scale depthwise convolutions into successive ConvNeXt-Tiny stages, which enables progressive refinement of contextual representations across the hierarchical feature levels in retinal OCT images.

### 2.3 Attention mechanisms and feature recalibration

Attention mechanisms are used in medical imaging for better feature representation. Channel attention methods such as Squeeze and Excitation networks (Hu et al., 2018) modify feature responses using global pooling and learned weights. Spatial attention highlights disease relevant regions. Hybrid methods like CBAM (Woo et al., 2018), utilize both channel and spatial cues together.

Attention based models in retinal OCT classification improve localization of disease specific regions, especially for subtle or low contrast abnormalities (Huang et al., 2023; Cao et al., 2024). Transformer based architectures, including efficient Vision Transformer variants such as EfficientViT (Liu et al., 2023) and hierarchical models such as Swin Transformer (Liu et al., 2021), extend this concept by modeling global relationships via self attention.

The attention techniques are mainly used as a feature weighting technique and not as a way of capturing multi-level context information explicitly. The transformer-based techniques tend to consume more computational resources and need large amounts of data for achieving desired performance. This makes them less suitable for deployment in clinical systems.

### 2.4 Modernized convolutional architectures in medical imaging

ConvNeXt (Liu et al., 2022) is a modern architectural approach to convolutional networks based on transformer models but still incorporating the inductive biases of convolutional neural networks. This model incorporates large kernel depthwise convolution, inverted bottlenecks, and layer normalization, thus improving feature extraction and training process. This combination provides Imagenet results comparable to other approaches in terms of parameter efficiency.

The different ConvNeXt architectures are widely used in various medical imaging scenarios owing to their stability and computational efficiency (Liu et al., 2022). Of all the architectures, the ConvNeXt-Tiny variant maintains a good trade-off between performance and computational costs.

The architecture of standard ConvNeXt models does not inherently include adaptive multi-scale context-aware modeling at different intermediate layers. The role of large convolutions is to widen the receptive field; however, they are unable to combine information on multiple dilations that would be suited for the variability of lesions. This limitation motivates architectural extensions designed specifically for spatially heterogenous medical imaging tasks.

Standard ConvNeXt architectures do not explicitly incorporate adaptive multi scale contextual modeling across intermediate stages. Although large convolutional kernels increase the receptive field, they do not dynamically combine information from multiple dilation scales in response to lesion variability. This limitation motivates development of architectural extensions designed specifically for spatially heterogeneous medical imaging tasks.

### 2.5 Reliability, uncertainty, and robustness in clinical AI systems

Accuracy in classification is a very popular metric for evaluating the performance of a model. But for the actual implementation of the model in the clinical setting, it is equally important to have reliable probability estimates and robustness to variations in image acquisition. Deep neural networks are known to be overconfident, i.e., to assign high probability to wrong predictions. This issue is addressed by calibration metrics, including Expected Calibration Error (ECE) and Brier score, which assess the correspondence between predicted probabilities and actual outcomes (Guo et al., 2017; Ovadia et al., 2019).

*Post hoc* temperature scaling has become one of the efficient and widely used methods for enhancing model calibration (Guo et al., 2017). Monte Carlo Dropout (Gal and Ghahramani, 2016) provides an approximate Bayesian approach for estimating predictive uncertainty by introducing stochasticity during inference. It enables analysis of prediction variability associated with model uncertainty.

Robustness to noise, blurring and changes in illumination is especially critical for OCT imaging because of variations in the instrument as well as errors during data acquisition. The statistical significance of performance improvements is evaluated using McNemar's test against the baseline ConvNeXt-Tiny model. Consequently, model reliability is not always examined in a manner that adequately supports practical clinical deployment.

### 2.6 Positioning of the proposed approach

Although there are still advancements being made in the field, retinal OCT classification has several difficulties. Many of the convolutional neural networks do not include information from multi-scale features during computation, possibly hindering their ability to encode various patterns. At the same time, improvements in the system's performance is achieved with attention based mechanisms and transformer based approaches, which increase the amount of computational load. In addition, evaluation is commonly centered on classification performance, while aspects such as predictive calibration and robustness under varying imaging conditions remain insufficiently explored.

To address these limitations, the proposed approach integrates the SAMC module with the ConvNeXt-Tiny backbone as a lightweight stage-wise refinement mechanism. The design builds on established principles of dilated convolution and multi-scale feature fusion, while adapting their configuration to successive ConvNeXt stages. Parallel depthwise convolutions extract complementary spatial contexts, and pointwise fusion combines the resulting representations before further hierarchical processing. Contextual information at multiple scales is extracted using dilated depthwise convolutions simultaneously, while adaptive fusion of features helps in fusing complementary information generated during intermediate layers of the network. In addition to the classification performance, the model is evaluated by calibration analysis, uncertainty estimation, robustness evaluation and statistical significance testing. These additional evaluations lead to a better understanding of the model behavior and a more reliable evaluation of the retinal OCT classification performance.

## 3 Materials and methods

### 3.1 Dataset description

The experiments were done on the OCT-C8 dataset (Naren, 2021). It has high resolution cross sectional retinal images from eight categories: normal retina, AMD, CSR, DME, DR, drusen, CNV, and MH. Each category includes an equal number of samples. The class distribution is balanced to allow regular training and neutral evaluation for each class.

The dataset consists of fixed image-level subsets for training (18,400 images), validation (2,800 images), and testing (2,800 images). Each subset includes eight class-specific folders corresponding to various retinal conditions. The distribution of classes is summarized in Table 2.

**Table 2:** OCT-C8 dataset sample distribution for training, validation and test sets.

Class	Train	Validation	Test	Total images
CSR	2,300	350	350	3,000
AMD	2,300	350	350	3,000
DME	2,300	350	350	3,000
CNV	2,300	350	350	3,000
DRUSEN	2,300	350	350	3,000
DR	2,300	350	350	3,000
MH	2,300	350	350	3,000
NORMAL	2,300	350	350	3,000
**Total**	**18,400**	**2,800**	**2,800**	**24,000**

Bold values indicate the total number of samples in each dataset split.

The OCT-C8 dataset offers pre-defined partitions for image-level training, validation, and testing. However, since the publicly available dataset lacks patient identifiers or subject-level information, it is impossible to verify patient-level separation. Consequently, the experiments conducted in this study adhere to the original image-level split provided by the dataset, without any additional reorganization. This evaluation protocol was chosen for the sake of consistency with previous benchmark studies on OCT-C8 and to enable fair comparison with existing methods for retinal OCT classification.

### 3.2 Data preprocessing and augmentation

All OCT images were preprocessed and augmented with a standardized pipeline prior to model training and dataset quality control. The first step was quality control of the dataset to ensure the integrity of the data. This included automatic checking of image readability during data loading to detect corrupted or unreadable files. Any samples that were not loaded correctly were filtered out of the training pipeline. The OCT-C8 dataset is a curated benchmark dataset with standardized class-wise organization, and no corrupted or invalid images were detected during this process.

All OCT images were then rescaled to a spatial dimension of 224 × 224 pixels to conform to the ConvNeXt-Tiny input dimensions and normalized using ImageNet statistics.

For training, spatial and photometric augmentations were applied to reflect variability in OCT image acquisition (Shorten and Khoshgoftaar, 2019; Buslaev et al., 2020). These included horizontal flipping, brightness and contrast adjustment, affine transformations (shift, scale, and rotation), grid distortion, elastic deformation, Gaussian blur, and coarse dropout to improve robustness. During validation and testing, only resizing and normalization were applied, with all augmentation operations disabled to ensure standardized evaluation without distributional distortion.

Only resizing and normalization were performed during validation and testing to ensure a standardized evaluation process without introducing extra perturbations. The training-time augmentation techniques, their categories, probability values, and corresponding objectives are summarized in Table 3.

**Table 3:** Training time augmentation strategy and functional objectives.

Transformation	Type	Probability	Objective
Horizontal flip	Geometric	0.5	Lateral invariance
Random brightness/Contrast	Photometric	0.6	Illumination robustness
Shift–scale–rotate	Geometric	0.5	Affine robustness
Grid distortion	Spatial	0.3	Structural deformation modeling
Elastic transform	Spatial	0.2	Non-linear variation simulation
Gaussian blur	Photometric	0.3	Noise robustness
Coarse dropout	Regularization	0.5	Occlusion tolerance
Normalization (ImageNet)	Preprocessing	1.0	Pre-trained weight compatibility

### 3.3 Cross-dataset leakage verification

To ensure strict separation between training and evaluation data, a cross-dataset leakage verification procedure was performed. In this process, each image from the OCT-C8 training set and the external Kermany OCT2017 Version 2 dataset obtained from the Kaggle repository was resized to 64 × 64 pixels and converted to grayscale. The external evaluation set consisted of 968 images from four classes (CNV, DME, DRUSEN, and NORMAL), with 242 images per class. A deterministic MD5 hash was computed from the flattened pixel intensity values of each image to generate a unique fingerprint. The resulting hash values were compared between datasets and images with the same hashes were considered duplicates. Note that this procedure was only designed for verification at evaluation time, and was not used during model training or preprocessing. We found no matching hash values between the OCT-C8 training set and the Kermany OCT2017 Version 2 test set, confirming that the datasets are strictly independent with no exact overlaps.

### 3.4 Baseline ConvNeXt architecture

**Figure 3:**
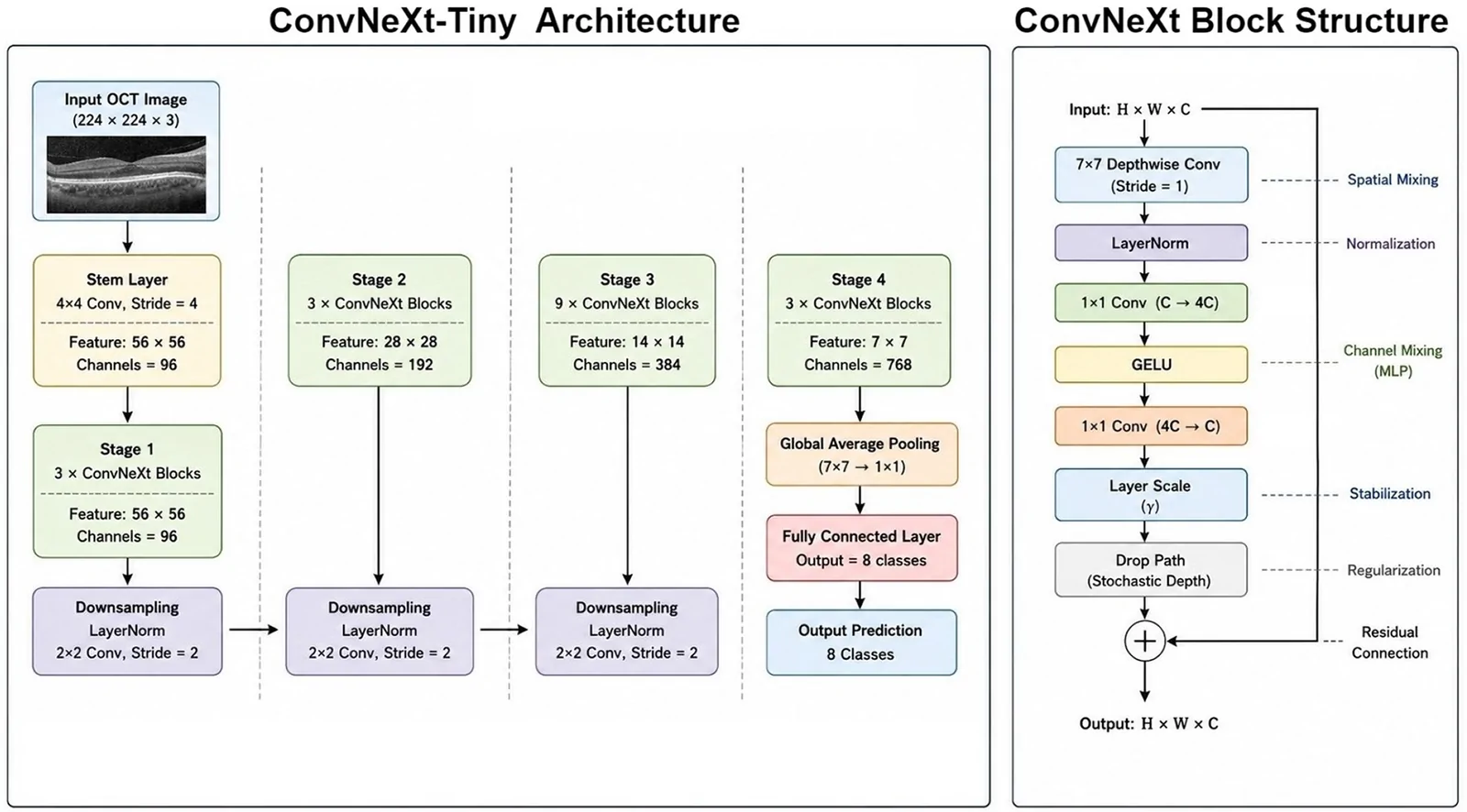
ConvNeXt-Tiny architecture and block structure showing the network pipeline and the ConvNeXt block.

In this work, we used ConvNeXt-Tiny as our baseline model. It is a modern convolutional architecture that takes design cues from vision transformers but still retains the inductive biases of convolutional networks. The network consists of four nested stages with channel sizes 96, 192, 384, and 768 and depth composition of (3, 3, 9, and 3) blocks in each stage. Figure 3 shows the entire layout of ConvNeXt-Tiny and its core block. Every block features the use of a huge kernel depthwise convolution of size (7 7). This operation is followed by layer normalization. The block also uses an inverted bottleneck with GELU activation and a residual connection. This allows both, effective spatial context modeling and efficient computing.

The backbone is initialized with the pre-trained weights of ImageNet 22K (Deng et al., 2009) to provide a solid basis for feature extraction and to improve the performance of transfer learning. The last classification layer was changed to give outputs for the eight retinal disease classes. A global average pooling layer was applied before applying the fully connected layer.

ConvNeXt-Tiny has around 28 million parameters and it strikes a good balance between representational capacity and computational cost, which makes it a good choice as the baseline for retinal OCT classification.

### 3.5 Proposed spatial adaptive multi-scale convolution module

**Figure 4:**
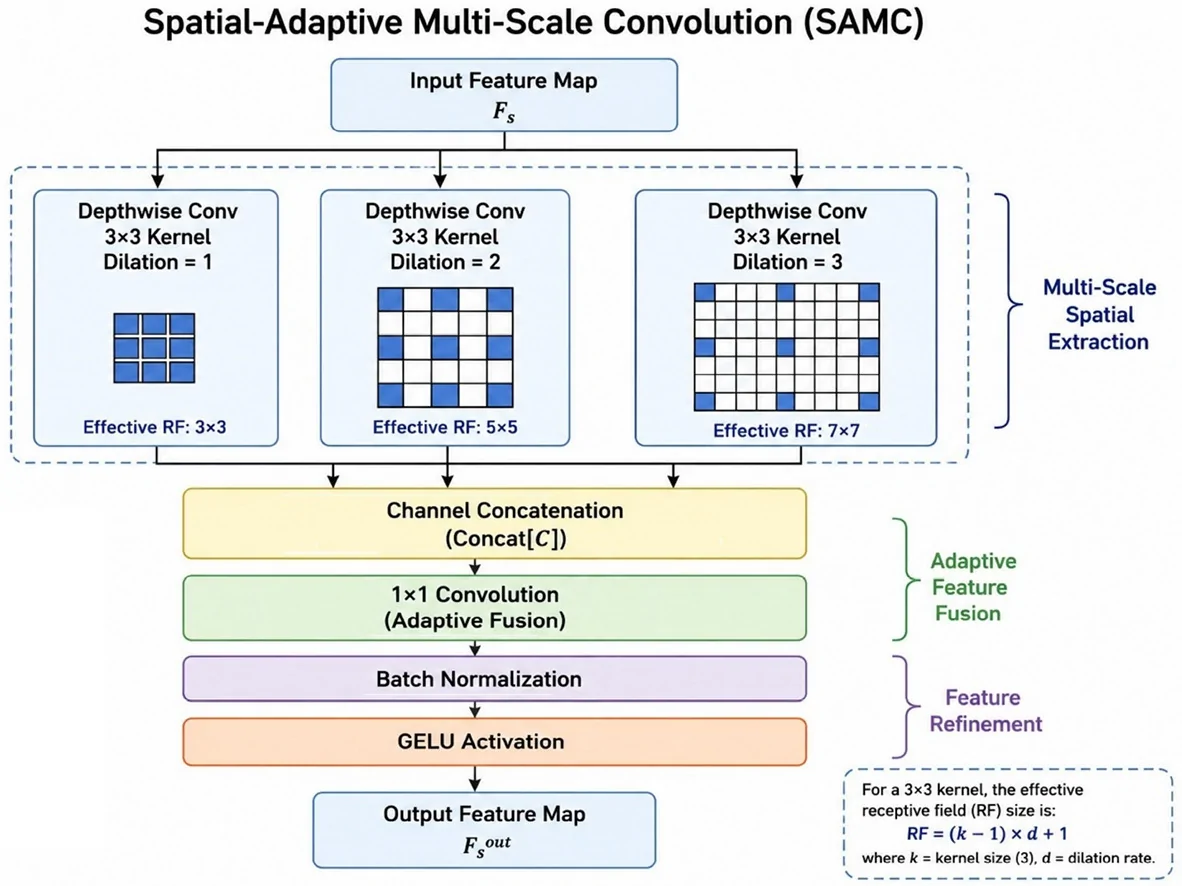
SAMC module architecture with parallel dilated convolutions and multi-branch feature integration.

Retinal OCT images not only include pathological patterns but these patterns can be found at very different spatial scales too. Small, localized abnormalities like drusen deposits might be present at the same times with very substantial structural changes, such as macular holes and fluid accumulations. To model this spatial variability explicitly, a Spatial Adaptive Multi-Scale Convolution (SAMC) module is devised and placed after every stage of the ConvNeXt backbone. The design of the SAMC module is shown in Figure 4.

Let ${\text{F}}_{s}\in {\mathbb{R}}^{C_{s}\times H_{s}\times W_{s}}$ denote the output feature map of stage *s*. The proposed SAMC module applies three parallel depthwise convolution branches with kernel size 3 × 3 and dilation rates *d* ∈ {1, 2, 3} (Yu and Koltun, 2015), as formulated in Equation 1:


$$\begin{array}{l} {\text{F}}_{s}^{\left(d\right)}={\text{DWConv}}_{3\times 3}^{\left(d\right)}\left({\text{F}}_{s}\right) \end{array}$$ (1)

Depthwise grouping (*groups* = *C*_*s*_) allows spatial filtering per channel with few extra parameters. Different dilation rates allow the model to extract local fine-grained features (*d* = 1) and contextual broad information (*d* = 2, 3) at the same time (Chen et al., 2018; Lin et al., 2017).

The resulting multi-scale representations are concatenated along the channel dimension (Gao et al., 2019), as expressed in Equation 2:


$$\begin{array}{l} {\text{F}}_{s}^{concat}=\text{Concat}\left({\text{F}}_{s}^{\left(1\right)},{\text{F}}_{s}^{\left(2\right)},{\text{F}}_{s}^{\left(3\right)}\right) \end{array}$$ (2)

A 1 × 1 pointwise convolution is subsequently applied to adaptively fuse the aggregated features, as defined in Equation 3:


$$\begin{array}{l} {\text{F}}_{s}^{fused}={\text{Conv}}_{1\times 1}\left({\text{F}}_{s}^{concat}\right) \end{array}$$ (3)

The fused features are passed through batch normalization followed by a GELU activation function, as described in Equation 4, to maintain stable training and incorporate non-linear transformations.


$$\begin{array}{l} {\text{F}}_{s}^{out}=\text{GELU}\left(\text{BN}\left({\text{F}}_{s}^{fused}\right)\right) \end{array}$$ (4)

The resulting feature map ${\text{F}}_{s}^{out}$ replaces the original stage output and propagated to the next downsampling layer. Unlike conventional static multi-branch fusion methods applied at a single network depth, SAMC is integrated after each ConvNeXt hierarchical stage, progressively refining contextual representations across expanding receptive fields. This configuration is intended to support the discrimination of retinal pathologies appearing at different anatomical scales.

With the improved contextual modeling capability, SAMC adds little computational overhead, which is a lightweight depthwise convolution and one 1 × 1 fusion layer in each stage. The proposed design avoids heavy attention or transformer-based self-attention mechanisms and keeps modest parameter growth while preserving computational efficiency.

#### 3.5.1 Comparison with existing multi-scale feature modeling strategies

Multi-scale feature modeling has been studied in several forms such as Atrous Spatial Pyramid Pooling (ASPP) in DeepLab (Chen et al., 2018) and Feature Pyramid Networks (FPN) (Lin et al., 2017). ASPP captures contextual information effectively using parallel atrous convolutions with different fixed dilation rates. For a given input feature representation *F*, the ASPP formulation is given in Equation 5:


$$\begin{array}{l} F_{ASPP}=Concat\left(Conv^{d_{1}}\left(F\right),Conv^{d_{2}}\left(F\right),...,Conv^{d_{n}}\left(F\right)\right) \end{array}$$ (5)

where *d*_*i*_ is the predefined dilation rate for capturing different receptive fields. ASPP is effective to enhance contextual coverage but it is usually operating on a single feature resolution and lacks the progressive representation refinement across different hierarchical stages of the backbone.

FPN uses a top-down pathway to combine feature maps of different backbone levels to aggregate multi-scale features. The feature fusion process is formulated in Equation 6:


$$\begin{array}{l} P_{l}=Conv_{1\times 1}\left(C_{l}\right)+Upsample\left(P_{l+1}\right) \end{array}$$ (6)

where *C*_*l*_ denotes the feature map from backbone stage *l*, and *P*_*l*_ denotes the fused pyramid representation. FPN effectively fuses semantic information across resolutions but its main operation is feature-level fusion rather than spatial contextual refinement at each backbone stage

In contrast, the proposed SAMC module performs stage-wise multi-scale spatial refinement in the hierarchical ConvNeXt backbone. SAMC takes an input feature representation *F*_*s*_ and applies depthwise dilated convolutions with several dilation rates, as defined in Equation 7:


$$\begin{array}{l} F_{s}^{d_{i}}=DWConv_{3\times 3}^{d_{i}}\left(F_{s}\right) \end{array}$$ (7)

where *d*_*i*_ ∈ {1, 2, 3} is the dilation setting employed in the proposed framework. The resulting multi-scale features are concatenated and adaptively fused according to Equation 8:


$$\begin{array}{l} F_{SAMC}=Conv_{1\times 1}\left(Concat\left(F_{s}^{d_{1}},F_{s}^{d_{2}},F_{s}^{d_{3}}\right)\right) \end{array}$$ (8)

Unlike ASPP that expands the receptive field in parallel at one feature level, and FPN that emphasizes hierarchical feature fusion, SAMC progressively improves the spatial contextual representations after each ConvNeXt stage. The proposed stage-wise integration is effective for multi-scale feature enhancement, while maintaining the compatibility with the lightweight hierarchical design of the ConvNeXt backbone.

### 3.6 Proposed ConvNeXt–SAMC architecture

To enhance spatial feature representation for retinal OCT image classification, the proposed Spatial Adaptive Multi-Scale Convolution (SAMC) module is integrated into the ConvNeXt-Tiny backbone.

**Figure 5:**
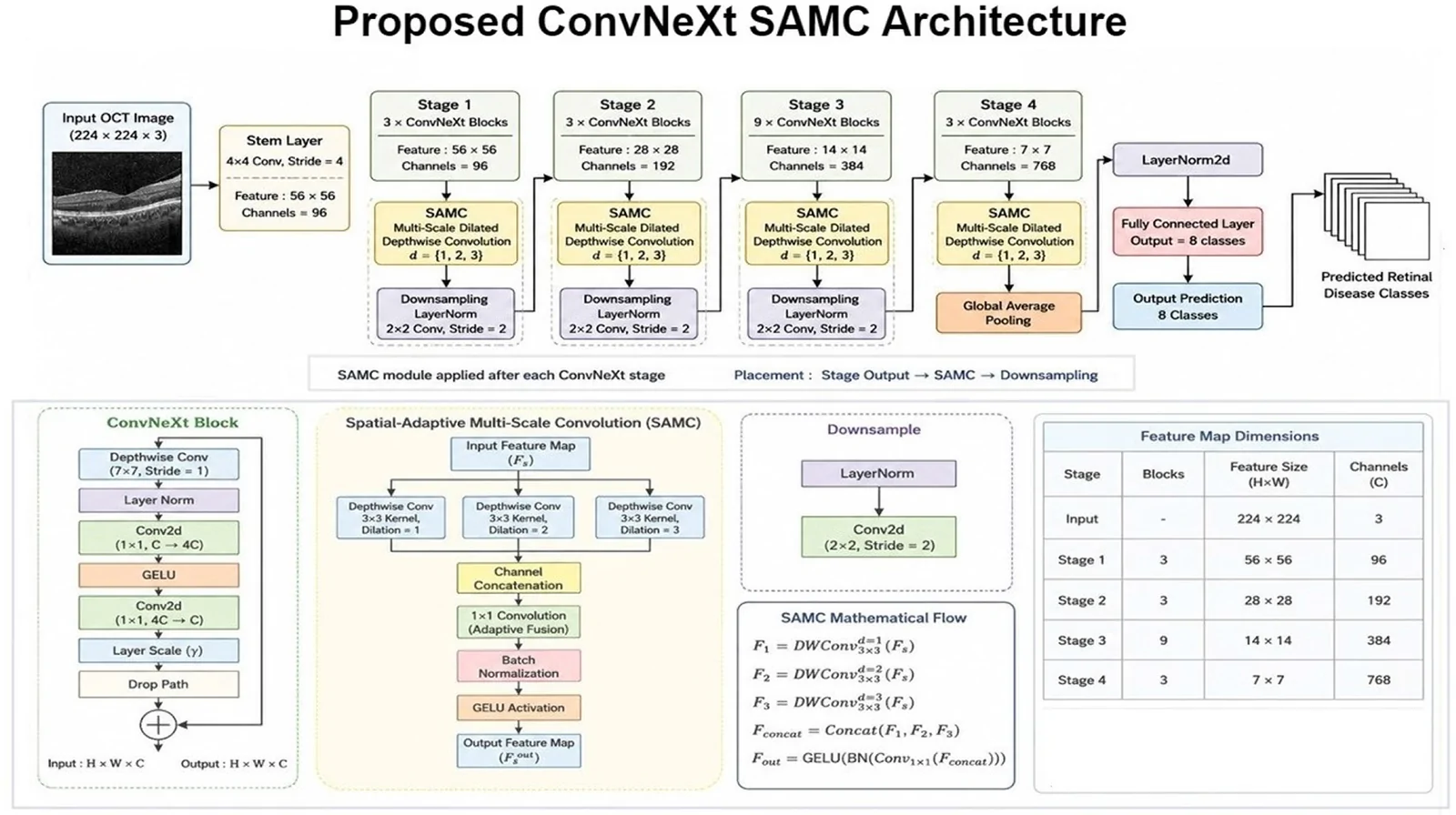
Overview of the proposed ConvNeXt-SAMC architecture for retinal OCT classification.

As illustrated in Figure 5, the proposed architecture extends the baseline ConvNeXt-Tiny model by integrating the SAMC module after each hierarchical stage of the network. The backbone performs progressive feature extraction through four stages with channel dimensions of 96, 192, 384, and 768. This enables representation learning at increasing semantic levels.

The ConvNeXt stages are formed by stacked ConvNeXt blocks consisting of depthwise convolution, layer normalization, channel expansion layers, GELU activation, and residual connections. This design supports computational efficiency while preserving rich stage level feature representations.

The SAMC module is used to improve the output of each stage before downsampling after feature extraction. SAMC utilizes three parallel depthwise convolution branches with the dilation rates of *d* = {1, 2, 3} to capture complementary spatial information in different receptive fields for each input feature map. The branch with *d* = 1 preserves fine local retinal structures, whereas larger dilation rates expand contextual coverage to model broader pathological patterns.

The multi-scale feature maps obtained are concatenated along the channel dimension and adaptively fused through a light weight 1 × 1 convolution. Its followed by batch normalization and GELU activation. Through this operation, refined contextual representations can be obtained while maintaining low computational complexity.

While traditional multi-branch fusion methods only operate at certain depths of the network, SAMC is implemented directly after each ConvNeXt stage. This way, contextual refinement can proceed progressively at all levels of the feature hierarchy. This stage wise design enables the network to capture both fine grained retinal textures and large scale anatomical variations at the same time. Thus, the model is more appropriate to identify retinal diseases that appear at different spatial scales and sizes.

After SAMC refinement, downsampling layers are applied to prepare the feature maps for the subsequent stage. Each downsampling block consists of LayerNorm, and a 2 × 2 convolution with stride 2. It reduces spatial resolution and increases channel dimensionality for the next stage. This hierarchical process is repeated across all four stages. This progressively transforms low level spatial features into high level semantic representations.

Finally, the output of the fourth stage is aggregated using global average pooling. It is followed by layer normalization. The normalized features are then passed to a fully connected classification layer. Predictions are then generated for the eight retinal disease classes.

Despite the additional contextual modeling capability, the proposed design remains computationally efficient because SAMC relies only on lightweight depthwise convolutions and a single 1 × 1 fusion operation per stage, avoiding the heavy overhead typically associated with attention-based or transformer-based contextual modules.

### 3.7 Training strategy

The proposed ConvNeXt-SAMC model was trained using a transfer learning (Pan and Yang, 2009) strategy to leverage pre-trained visual representations while adapting the network to the retinal OCT classification task. The backbone network was initialized with weights pretrained on the ImageNet-22K dataset, enabling the model to benefit from large-scale visual feature representations learned from natural images.

Optimization was performed using the AdamW (Loshchilov and Hutter, 2017) optimizer, which decouples weight decay from gradient updates and has been shown to improve generalization in modern deep neural networks. The initial learning rate was set to 1 × 10^−4^, with a weight decay of 1 × 10^−5^ to regularize model parameters and reduce overfitting. The complete training configuration and hyperparameter settings used in this study are summarized in Table 4.

**Table 4:** Training settings and hyperparameters of the ConvNeXt-SAMC model.

Parameter	Configuration
Hardware platform	NVIDIA Tesla T4 GPU (Google Colab)
Training precision	Mixed precision (FP16)
Batch size	32
Training epochs	80
Optimizer	AdamW
Initial learning rate	1 × 10^−4^
Loss function	Cross-entropy with label smoothing
Weight decay	1 × 10^−5^
Learning rate scheduler	Cosine annealing
Model selection criterion	Highest validation accuracy

To improve generalization and mitigate overconfident predictions, cross-entropy loss with label smoothing was employed. Label smoothing (Szegedy et al., 2016; Minderer et al., 2021) encourages the model to produce less overconfident probability estimates, which is particularly beneficial for medical decision-support systems where reliable confidence estimation is important.

Training uses a cosine annealing schedule (Loshchilov and Hutter, 2016), where the learning rate decreases progressively over epochs. This supports faster convergence at the beginning and more stable updates in later stages.

Mixed precision training (Micikevicius et al., 2017) is used over 80 epochs to reduce memory usage and improve efficiency. Model selection is based on validation accuracy, and the best performing checkpoint is retained. Gradient updates were performed with automatic differentiation frameworks and the checkpoints saved periodically throughout the training process.

### 3.8 Performance evaluation metrics

The effectiveness of the proposed framework was assessed using standard classification metrics (Powers, 2020; Sokolova and Lapalme, 2009; Fawcett, 2006) applied in medical image analysis. These metrics capture different aspects of predictive behavior and support assessment of diagnostic performance across all retinal disease categories.

In medical classification tasks, both false positive and false negative errors can have significant clinical consequences. Therefore, multiple evaluation metrics were considered to assess different aspects of model performance. The metrics used in this study include accuracy, recall, precision, specificity and F1 score.

Let *TN*_*c*_, *TP*_*c*_, *FN*_*c*_, and *FP*_*c*_ denote the number of true negatives, true positives, false negatives, and false positives for class *c*, respectively.

#### 3.8.1 Accuracy

Accuracy measures the overall proportion of correctly classified samples among all test samples. It gives a general measure of the model's ability to correctly identify both diseased and normal retinal conditions. Accuracy is calculated using Equation 9.


$$\begin{array}{l} \text{Accuracy}=\frac{\sum_{c}\left(TP_{c}+TN_{c}\right)}{\sum_{c}\left(TP_{c}+TN_{c}+FP_{c}+FN_{c}\right)} \end{array}$$ (9)

Although accuracy provides a useful summary of performance, it may not fully reflect model behavior when evaluating individual classes in multi class medical classification tasks.

#### 3.8.2 Precision

Precision is a measure of the correctness of the positive classifications. Higher precision means fewer false positive predictions. Precision is computed using Equation 10.


$$\begin{array}{l} {\text{Precision}}_{c}=\frac{TP_{c}}{TP_{c}+FP_{c}} \end{array}$$ (10)

This is important in medical imaging because false alarms may result in unnecessary clinical examinations and can increase healthcare costs.

#### 3.8.3 Recall (sensitivity)

Recall, also referred to as sensitivity, measures the proportion of actual positive cases that are correctly detected by the model. Recall is calculated using Equation 11.


$$\begin{array}{l} {\text{Recall}}_{c}=\frac{TP_{c}}{TP_{c}+FN_{c}} \end{array}$$ (11)

High recall is particularly important in disease detection tasks because failing to identify a pathological condition may delay treatment and negatively affect patient outcomes.

#### 3.8.4 F1-score

The F1 score combines precision and recall into a single metric through their harmonic mean. The F1-score is computed using Equation 12.


$$\begin{array}{l} {\text{F1}}_{c}=\frac{2TP_{c}}{2TP_{c}+FP_{c}+FN_{c}} \end{array}$$ (12)

It is helpful when reducing both false positives and false negatives is important.

#### 3.8.5 Specificity

Specificity measures the proportion of correctly identified negative cases among all actual negative samples. Specificity is calculated using Equation 13.


$$\begin{array}{l} {\text{Specificity}}_{c}=\frac{TN_{c}}{TN_{c}+FP_{c}} \end{array}$$ (13)

A high specificity value indicates fewer false positive classifications.

Each of the eight retinal diseases categories was separately evaluated. Then the results were macro averaged to get an overall performance summary. This method allows equal weight to be given to each class and a single class will not dominate the evaluation. Further, it ensures that different disease categories are uniformly evaluated. Both per class results and macro averaged metrics are given in the results section to show complete diagnostic performance evaluation.

### 3.9 Reliability and calibration analysis

In clinical decision support systems, the availability of reliable probability estimates is crucial. Yet, deep neural networks sometimes produce confident predictions even when they are wrong, which is a case of miscalibration. This is a serious issue in medical imaging, as the confidence level output by the model may not indicate the actual probability of being correct.

Calibration measures are used to measure the accuracy with which predictions match with actual results for the proposed model. ECE and Brier score (Guo et al., 2017; Brier, 1950; Zhu et al., 2025) are used to assess prediction calibration.

#### 3.9.1 Expected calibration error

ECE tries to quantify how much confidence scores that are predicted deviate from the actual accuracy. First, the predictions are divided into M intervals of confidence levels, then the difference is calculated between the mean confidence and accuracy within each interval. The Expected Calibration Error (ECE) is calculated using Equation 14.


$$\begin{array}{l} ECE=\overset{M}{\underset{m=1}{\sum }}\frac{n_{m}}{N}|{\text{Acc}}_{m}-{\text{Conf}}_{m}| \end{array}$$ (14)

where *n*_*m*_ is the number of samples in bin *m*, and *N* is the total number of samples. Acc_*m*_ and Conf_*m*_ denote the accuracy and average confidence of bin *m*, respectively.

Lower ECE values indicate better calibration, meaning the predicted probabilities closely reflect the true likelihood of correctness.

#### 3.9.2 Brier score

The Brier Score measures the mean squared difference between predicted probabilities and the actual class labels. It evaluates both the accuracy and calibration of probabilistic predictions. The Brier score is computed using Equation 15.


$$\begin{array}{l} \text{Brier}=\frac{1}{N}\overset{N}{\underset{k=1}{\sum }}\overset{K}{\underset{j=1}{\sum }}{\left({\hat{p}}_{k}^{\left(j\right)}-y_{k}^{\left(j\right)}\right)}^{2} \end{array}$$ (15)

where *N* denotes the total number of samples, and *K* is the number of classes. ${\hat{p}}_{k}^{\left(j\right)}$ represents the predicted probability for class *j* of sample *k*, while $y_{k}^{\left(j\right)}$ is the corresponding ground truth indicator.

Lower Brier scores indicate better probabilistic prediction quality.

#### 3.9.3 Temperature scaling for calibration

To improve the calibration of the predicted probabilities, temperature scaling was applied (Guo et al., 2017) as a post-processing technique. Temperature scaling adjusts the confidence of the softmax output using a temperature parameter *T*. Temperature scaling is performed using Equation 16.


$$\begin{array}{l} {\tilde{p}}_{j}=\frac{\exp\left(z_{j}/T\right)}{\sum_{l=1}^{K}\exp\left(z_{l}/T\right)} \end{array}$$ (16)

where *z*_*j*_ represents the logit corresponding to class *j*. The temperature parameter *T* is optimized on a validation set to reduce calibration error without affecting classification accuracy.

#### 3.9.4 Uncertainty estimation using Monte Carlo Dropout

In addition to calibration analysis, predictive uncertainty was estimated using Monte Carlo Dropout (Gal and Ghahramani, 2016). During stochastic inference, dropout layers were explicitly activated with a dropout probability of 0.2, while the remaining network components were maintained in evaluation mode to preserve learned normalization statistics.

For each test sample, 50 stochastic forward passes were performed through the network. The mean of the generated probability distributions was considered the final prediction probability, while the variance across stochastic predictions was used to quantify predictive uncertainty.

In this study, MC Dropout is applied as an uncertainty estimation method to analyze predictive variability during stochastic inference. The estimated uncertainty provides additional information about prediction stability and does not represent a modification of the underlying classification architecture.

Calibration and uncertainty analyses are performed to characterize the probabilistic behavior of the trained ConvNeXt-SAMC model. The related observations are given in Section 4.

### 3.10 Robustness testing

Performance of the model in real-world applications might differ from that in ideal settings. OCT images acquired in clinical practice usually suffer from certain flaws caused by the presence of noise, motion during image acquisition, variance of the devices, and variability of lighting conditions. All these elements may affect the predictions of the model. Thus, to examine the behavior stability of the ConvNeXt-SAMC model in such circumstances, the latter was subjected to the examination under the mentioned changes. The trained model was analyzed using perturbed data samples taken from the initial test dataset. Perturbation techniques were applied to reproduce the mentioned issues occuring during the OCT data acquisition process (Hendrycks and Dietterich, 2019; Ovadia et al., 2019; Rasti et al., 2017). The model was evaluated using Gaussian noise, blur, and brightness changes, which are commonly observed in OCT imaging.

#### 3.10.1 Gaussian noise

Gaussian noise was introduced to the test images to mimic the sensor noise and acquisition artifacts typically connected to medical imaging devices. Noise samples were drawn from a Gaussian distribution. Different standard deviation values were employed to produce multiple levels of image corruption. The retinal structure was maintained throughout this procedure.

#### 3.10.2 Gaussian blur

Gaussian blur was applied to simulate defocus and motion related artifacts that may occur during image acquisition. This perturbation reduces high frequency details in OCT scans. As a result, fine spatial information becomes partially degraded. The resulting blurred images were then analyzed using the proposed model to determine its ability to detect abnormal structures under such blurred images.

#### 3.10.3 Brightness variation

Brightness variations were done to mimic the effect of variable light and device specific imaging conditions. The pixel intensities in the images were changed within a limited range so that it could be tested if the model output is still unchanged when the lighting conditions are different.

For each perturbation type, the classification performance of the proposed ConvNeXt–SAMC model was evaluated using the same performance metrics described in Section 3.7. By comparing the model performance on perturbed images with the baseline test results, the robustness of the proposed framework under non-ideal imaging conditions can be quantitatively assessed.

### 3.11 Statistical significance analysis

To verify whether the performance improvement achieved by the proposed ConvNeXt–SAMC model over the baseline ConvNeXt architecture is statistically significant, McNemar's test was performed (McNemar, 1947; Dietterich, 1998; Demšar, 2006). McNemar's test is a non-parametric statistical test commonly used to compare the predictive performance of two classification models evaluated on the same test dataset.

The test analyzes the disagreement between two classifiers by examining the number of samples misclassified differently by each model. Let *n*_01_ denote the number of samples correctly classified by the baseline model but misclassified by the proposed model, and *n*_10_ denote the number of samples correctly classified by the proposed model but misclassified by the baseline model.

The McNemar test statistic is computed according to Equation 17:


$$\begin{array}{l} \chi^{2}=\frac{{\left(|n_{01}-n_{10}|-1\right)}^{2}}{n_{01}+n_{10}} \end{array}$$ (17)

where the correction term improves the accuracy of the test for finite sample sizes.

The null hypothesis assumes that both models have equal classification performance, meaning that the probability of disagreement in either direction is the same. A p-value is computed from the chi-square distribution with one degree of freedom.

A 95% confidence threshold (α = 0.05) is used as the decision criterion. Differences associated with *p*-values below this threshold were treated as unlikely to arise from random variation, indicating a meaningful performance gap between the ConvNeXt-SAMC and baseline ConvNeXt model.

McNemar test results and corresponding p-values are reported in the Results section, along with their impact on performance differences.

## 4 Results

### 4.1 Overall classification performance

A separate test set of 2,800 OCT images is used for evaluation, with samples evenly distributed across the eight retinal categories. The proposed model achieves a test accuracy of **98.39%** and a macro F1 score of **0.9839**, demonstrating consistent performance across all classes.

Reliability is examined using bootstrap-based confidence intervals at the 95% level. The estimated ranges are:

- Accuracy: 0.9841 (95% CI: [0.9789–0.9886])
- Macro F1-score: 0.9839 (95% CI: [0.9792–0.9883])

Confidence intervals are consistent across all samples, which implies stable behavior. Class-wise performance results can be found in Table 5. Perfect classification accuracy is recorded in the DR, AMD, MH, and CSR classes. All of these classes have an F1 score of 1.00.

**Table 5:** Per-class performance of the ConvNeXt-SAMC model on the OCT-C8 test dataset.

Class	Recall	F1-score	Precision
CSR	1.0000	1.0000	1.0000
AMD	1.0000	1.0000	1.0000
DR	1.0000	1.0000	1.0000
MH	1.0000	1.0000	1.0000
DME	0.9571	0.9682	0.9795
CNV	0.9657	0.9657	0.9657
DRUSEN	0.9600	0.9628	0.9655
NORMAL	0.9886	0.9746	0.9611
**Macro Avg**	**0.9839**	**0.9839**	**0.9840**

Bold values indicate the macro-average performance across all classes.

Performance differences were evident among the classes, Normal, CNV, DRUSEN, and DME. The four classes show some degree of overlap in terms of their morphological appearance in the OCT image. Differentiation is thus difficult to accomplish even by a experienced clinicians. Despite such conditions, accuracy, F1 score, and recall were relatively high in all the classes.

**Figure 6:**
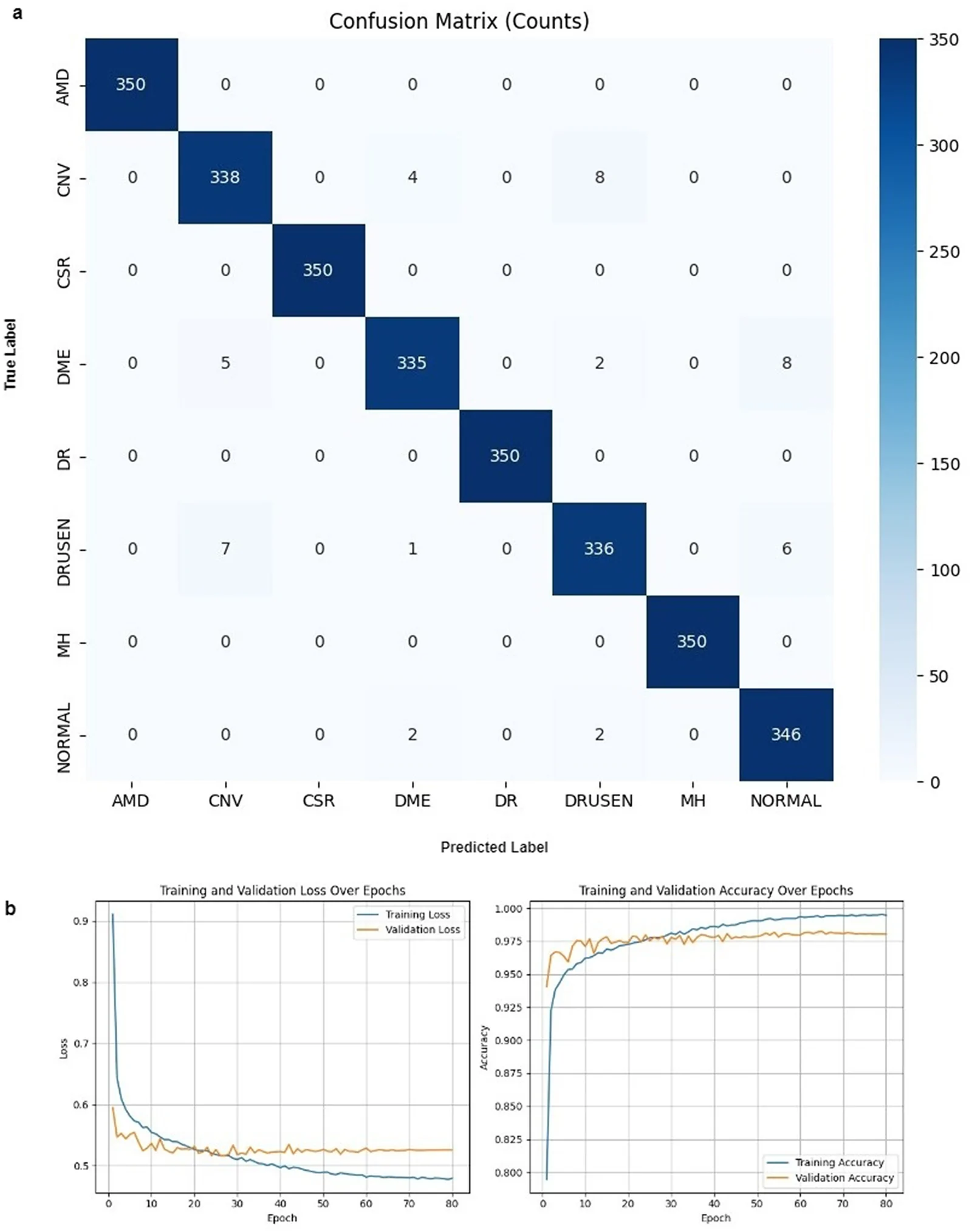
Classification performance and convergence behavior of the proposed ConvNeXt-SAMC model. **(a)** Confusion matrix obtained on the OCT-C8 test dataset, showing class-wise prediction outcomes across eight retinal disease categories. **(b)** Training and validation loss and accuracy curves over 80 epochs, demonstrating stable convergence and generalization capability.

The confusion matrix of the proposed ConvNeXt-SAMC model is presented in Figure 6a, demonstrating class-wise prediction performance across the eight retinal disease categories. The confusion matrix illustrates that most test samples were classified correctly. A perfect classification rate was obtained for the classes of DR, AMD, MH, and CSR, having 350 correctly classified samples in each.

Only a few errors were seen, mostly among DRUSEN, CNV, DME, and NORMAL. There were some CNV data included in DRUSEN category, and there were some DME data included in either CNV or NORMAL classes. This kind of confusion is inevitable since these conditions share similar structures in OCT scans.

The macro-specificity score was estimated at 0.9977, which shows that the model produced very few false positives.

### 4.2 Convergence behavior

The training and validation curves demonstrate that the proposed ConvNeXt-SAMC model achieved stable convergence without divergence or significant training instability. The best accuracy achieved was 98.25% at epoch 65 for the 80-epoch training. The training loss decreases and the validation loss stabilizes gradually after the initial training period, which indicates that the optimization and feature learning are effective.

The highest validation accuracy achieved during the 80-epoch training process was 98.25% at epoch 65. The training loss exhibited a continuous decreasing trend, while the validation loss gradually stabilized after the initial training phase, indicating effective optimization and improved feature learning.

Figure 6b shows the corresponding training and validation loss and accuracy curves. As shown in Figure 6b, the model had a growing trend of accuracy in training and then stabilized in the later epochs. The stable performance of the model on both training and validation datasets suggests that the model has a good generalization ability and does not overfit. The obtained results demonstrate the effectiveness of the proposed training strategy and the ability of the ConvNeXt-SAMC architecture to learn discriminative features for multi-class retinal OCT classification.

### 4.3 Calibration and reliability analysis

In addition to the assessment of the effectiveness of classification performance, the proposed model was evaluated for the consistency of probability prediction using calibration measures. In medical imaging, the decision making can be affected by any inaccuracy in probability estimation, thus the accurate probability estimates are crucial.

For deterministic inference, the proposed ConvNeXt-SAMC model achieved an Expected Calibration Error (ECE) of 0.0785 and a Brier score of 0.0338. ECE was reduced to 0.0386 and Brier score was reduced to 0.0290 after temperature scaling. Temperature scaling impacts the confidence calibration but the predicted class labels remain unchanged so the classification accuracy did not change.

After training we also apply temperature scaling as an additional calibration method. The optimal temperature parameter determined by validation was *T* = 0.858. After applying temperature scaling, there was a significant improvement in calibration results. The ECE measure became 0.0386 (as compared with 0.0785), while the Brier score dropped to 0.0290.

**Figure 7:**
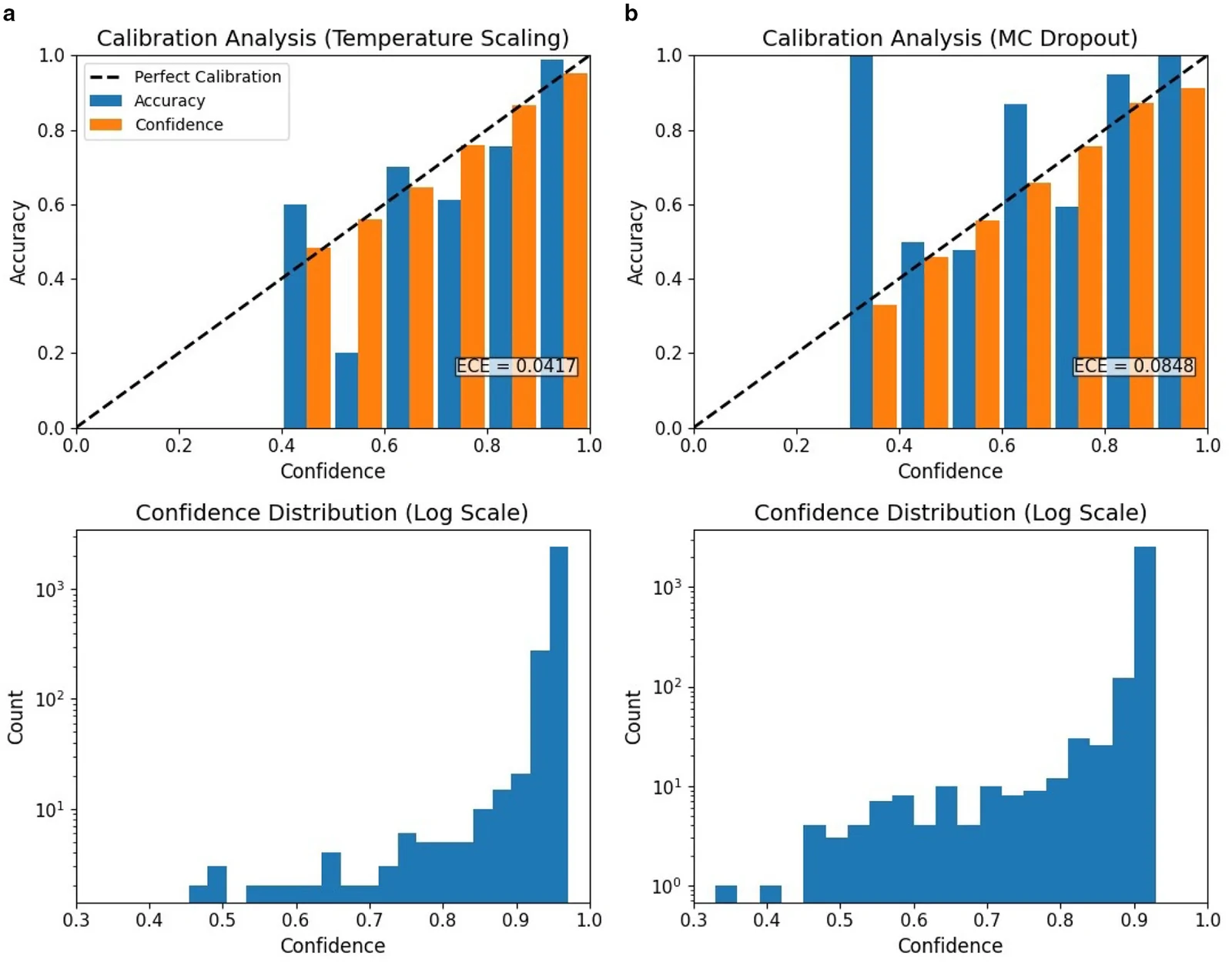
Calibration analysis of the proposed ConvNeXt-SAMC model using **(a)** temperature scaling and **(b)** Monte Carlo Dropout-based stochastic inference. The top row presents reliability diagrams comparing predicted confidence with observed accuracy, where the dashed diagonal indicates perfect calibration. The bottom row presents confidence distributions on a logarithmic scale. MC Dropout uncertainty estimation was performed using a dropout probability of 0.2 with 50 stochastic forward passes.

Figure 7 demonstrates the calibration performance of the ConvNeXt-SAMC model in the context of temperature scaling and Monte Carlo dropout. The reliability graphs suggest that the true accuracy values inside each confidence interval are consistent with the respective probability predictions. The predicted probabilities show reasonable agreement with observed outcomes, with further calibration improvement obtained through temperature scaling. The confidence values are highly skewed toward the more confident predictions. Even with this skew, no substantial overconfidence is observed, as calibration remains stable across intervals that contain sufficient samples. The concentration of samples in high confidence bins results in limited representation of lower confidence intervals, which may lead to less smooth calibration profiles despite low calibration error.

Besides calibration analysis, predictive uncertainty was evaluated using Monte Carlo Dropout. After enabling stochastic inference with a dropout probability of 0.2 and performing 50 forward passes per test sample, the MC Dropout evaluation produced an ECE value of 0.0848 and a Brier score of 0.0348. The mean predictive variance obtained from stochastic predictions was 0.000643, indicating measurable prediction variability while maintaining overall prediction stability.

Unlike temperature scaling, which directly improves probability calibration, MC Dropout does not modify the predicted class labels. Instead, it provides additional information regarding predictive uncertainty and confidence variability of the trained ConvNeXt-SAMC model.

### 4.4 Robustness evaluation

The robustness of the proposed ConvNeXt-SAMC model was evaluated under controlled image perturbations commonly encountered in clinical OCT imaging, including Gaussian noise, Gaussian blur, and brightness variations. The quantitative robustness results under different perturbation conditions are summarized in Table 6. The proposed model maintains stable classification performance under Gaussian noise, Gaussian blur, and brightness variations, demonstrating robustness against common OCT image degradations. The maximum accuracy drop under Gaussian noise is 0.07% at σ = 0.05. Similarly, Gaussian blur did not affect accuracy much, with accuracy remaining above 98% even for the largest blur kernel. The variations in brightness resulted in minor changes in the classification accuracy, showing that the method is robust to variations in illumination during the image acquisition. The slight improvements under mild brightness variation and low level noise are due to the regularization effect, where small perturbations improve the generalization of features. The maximum deviation from the clean test accuracy (98.39%) was about 0.36% for all the perturbation conditions, which demonstrates the robustness of the proposed ConvNeXt-SAMC framework under the realistic OCT imaging conditions. Furthermore, calibration metrics [e.g., Expected Calibration Error (ECE) and Brier score] were unaffected by perturbations, suggesting stable confidence estimation in non-ideal imaging settings.

**Table 6:** Robustness evaluation of the proposed ConvNeXt-SAMC model under different image perturbations.

Perturbation type	Severity level	Accuracy (%)
Gaussian noise	σ = 0.01	98.43
	σ = 0.03	98.43
	σ = 0.05	98.32
Gaussian blur	Kernel size = 3	98.11
	Kernel size = 5	98.04
Brightness variation	−0.15	98.50
	+0.15	98.43

#### 4.4.1 Per-class robustness analysis

To further understand the robustness at the disease-category level, the variation of the per-class F1-score under heavy Gaussian noise (σ = 0.05) and Gaussian blur (kernel size = 5) was studied as well. This analysis provides insight into whether or not certain categories of retinal disease are consistently recognized in the face of degraded imaging conditions, as opposed to overall accuracy.

The proposed ConvNeXt-SAMC model exhibited stable classification performance over the eight retinal categories, as shown in Table 7. The mean F1-score reduced from 0.9839 under clean test conditions to 0.9828 under Gaussian noise and 0.9807 under Gaussian blur. The corresponding mean F1 degradation was only 0.0011 and 0.0032, respectively. The limited degradation observed in lesion-associated categories confirms that the proposed model preserves discriminative representations for small and subtle retinal abnormalities, which are generally more vulnerable to image degradation.

**Table 7:** Per-class F1-score robustness analysis under Gaussian noise and blur perturbations.

Class	Clean F1	Noise F1	ΔF1 noise	Blur F1	ΔF1 blur
AMD	1.0000	1.0000	0.0000	1.0000	0.0000
CNV	0.9657	0.9630	0.0028	0.9658	–0.0001
CSR	1.0000	1.0000	0.0000	1.0000	0.0000
DME	0.9682	0.9652	0.0030	0.9614	0.0068
DR	1.0000	1.0000	0.0000	1.0000	0.0000
DRUSEN	0.9628	0.9612	0.0016	0.9551	0.0076
MH	1.0000	1.0000	0.0000	1.0000	0.0000
NORMAL	0.9746	0.9734	0.0013	0.9633	0.0114
**Mean**	**0.9839**	**0.9828**	**0.0011**	**0.9807**	**0.0032**

ΔF1 represents the performance degradation compared with clean test conditions. Bold values indicate the mean performance across all classes.

Particularly, lesion-sensitive categories such as CNV, DME, and DRUSEN exhibited limited performance variation. Under blur perturbation, CNV showed no degradation, while DME and DRUSEN experienced F1 reductions of only 0.0068 and 0.0076, respectively. These results indicate that the multi-scale contextual refinement introduced by SAMC preserves discriminative pathological representations even when OCT image quality is affected by noise and loss of fine structural details.

### 4.5 Statistical significance analysis

To evaluate whether the performance improvement achieved by the proposed ConvNeXt-SAMC model over the baseline ConvNeXt architecture is statistically significant, McNemar's test was conducted using paired predictions on the test dataset. McNemar's test (*n*_01_ = 29, *n*_10_ = 12) yielded a statistic of 6.24 with a *p*-value of 0.0124. The results of the McNemar statistical test are summarized in Table 8.

**Table 8:** Statistical significance analysis using McNemar's test comparing the baseline ConvNeXt model and the proposed ConvNeXt-SAMC model.

Model comparison	*n*_01_	*n*_10_	McNemar statistic (χ^2^)	*p*-value
ConvNeXt vs ConvNeXt–SAMC	29	12	6.24	0.0124

The *p* value is under 0.05 therefore the null hypothesis is rejected. This shows that the proposed model achieves a statistically significant improvement over the baseline model. The result suggests that the performance gain obtained by integrating the SAMC module is unlikely to be due to chance. Furthermore, the ConvNeXt-SAMC model correctly classifies significantly more test samples than the baseline ConvNeXt model. The findings confirm the effectiveness of the proposed SAMC module in improving classification performance.

These findings indicate that the observed performance gain is unlikely to be due to random variation. Instead, it reflects a meaningful improvement introduced by the proposed SAMC module. The statistical validation provides additional evidence for the effectiveness of the ConvNeXt-SAMC framework. It also supports its suitability for reliable multi class retinal OCT classification.

### 4.6 Model interpretability analysis

#### 4.6.1 Grad-CAM visualization

The interpretability measure uses Grad-CAM for analysis. This technique highlights the parts of the image which have the most significant impact on the output of the network's predictions. The resulting heat maps provide an interpretation of the neural network operation, pointing to the areas on which the model focuses to assign a class label.

**Figure 8:**
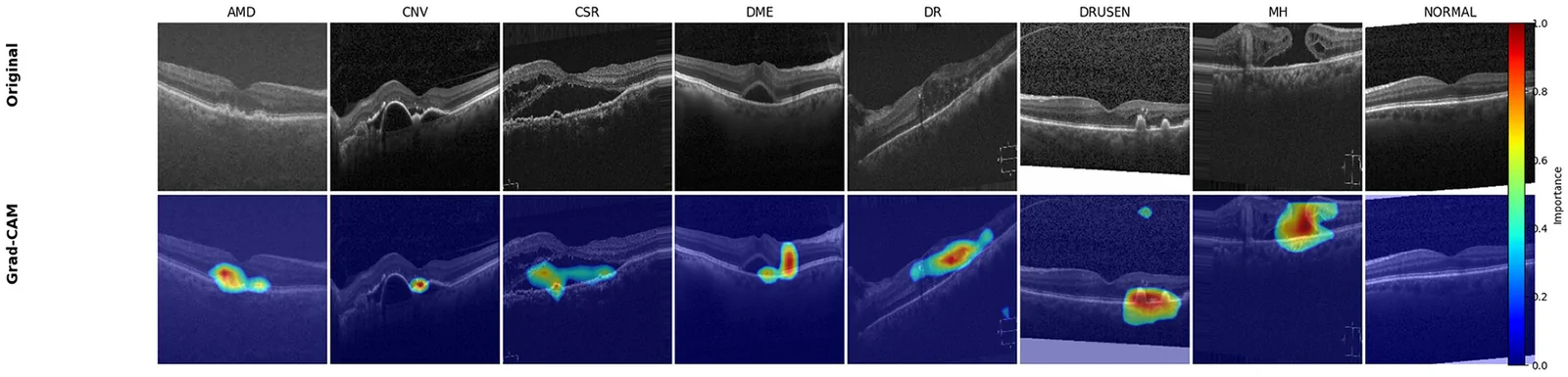
Grad CAM maps for 8 retinal OCT classes with the ConvNeXt-SAMC model. The **top** row shows the input images. The **bottom** row shows the corresponding activation maps. In CNV, DME, and MH, the responses are more localized. AMD and CSR have a larger distribution. DR and DRUSEN have smaller areas. LOW activation in NORMAL samples. The color bar indicates the level of contribution, where warmer colors are for higher values.

The Grad-CAM activation results were obtained from samples with high certainty in classification and were correctly classified. The figures for each class are shown in Figure 8. The activation maps of the model for the CNV and DME cases emphasize the regions related to fluid accumulation and abnormal vascular proliferation, respectively. The Grad-CAM activation of the MH samples is mainly focused on the foveal region.

AMD and CSR shows more spread across retinal layer with gradual layer variations. In DRUSEN, responses are more localized around deposit regions. In NORMAL samples, the activation is weaker and does not focus on irrelevant background areas.

A Grad-CAM map was generated using the final convolutional feature block to ensure that high-level discriminative regions were consistently visible.

Different samples exhibit comparable patterns. It is important to note that the regions that are highlighted are typically associated with visible structural changes and are not random or shifting activations.

#### 4.6.2 Disease-specific correspondence and quantitative analysis of Grad-CAM activation patterns

The regions that influence the ConvNeXt-SAMC predictions are visualized qualitatively in Figure 8. The clinical importance of the highlighted regions was further analyzed. A disease-specific correspondence analysis was conducted by comparing characteristic OCT abnormalities with observed Grad-CAM activation patterns.

The model exhibited anatomically plausible attention behavior in all eight retinal categories, as summarized in Table 9. AMD and CSR displayed broader activation in outer retinal and sub-retinal regions whereas CNV, DME, DRUSEN, and MH displayed more localized activation around disease associated structures. NORMAL samples showed no focal pathological attention and less intense and diffuse activation.

**Table 9:** Disease-specific qualitative correspondence between characteristic OCT features and Grad-CAM activation patterns.

Class	Characteristic OCT features	Observed Grad-CAM activation region	Qualitative correspondence
AMD	RPE irregularity, drusen-related elevation, and outer retinal disruption	Activation around abnormal outer retinal and RPE-associated regions	Consistent
CNV	Sub-retinal hyperreflective abnormalities, RPE disruption, and intraretinal/sub-retinal fluid	Focused activation around sub-retinal and RPE-associated abnormal regions	Consistent
CSR	Serous neurosensory retinal detachment and sub-retinal fluid accumulation	Broader activation over elevated retinal and sub-retinal fluid-associated regions	Consistent
DME	Intraretinal cystoid spaces and retinal thickening	Localized activation around cystic and thickened retinal regions	Consistent
DR	Retinal structural alterations associated with diabetic retinal changes	Activation over structurally abnormal retinal regions	Consistent
DRUSEN	Focal elevations involving the RPE–Bruch's membrane complex	Localized activation near outer retinal and RPE elevation regions	Consistent
MH	Foveal defect, retinal discontinuity, and surrounding retinal distortion	Focused activation around the foveal defect region	Consistent
NORMAL	Preserved retinal layer architecture	Low-intensity diffuse activation without focal pathological regions	No focal pathological activation

Since OCT-C8 provides only image-level labels and lacks pixel-level lesion annotations, direct lesion-overlap metrics such as Grad-CAM IoU or Dice coefficient cannot be computed. Therefore, the correspondence analysis represents qualitative agreement between model attention and known OCT characteristics rather than lesion localization accuracy.

To provide quantitative assessment, Grad-CAM statistics were calculated on 2,755 correctly classified test samples. The final ConvNeXt feature block was used as the target layer, and activation maps were normalized to [0,1]. Two complementary measures were calculated: activation coverage, defined as the percentage of pixels with activation values greater than 0.5, which quantifies the spatial extent of discriminative regions, and mean CAM intensity, which measures the overall strength of model attention. The class-wise results are presented in Table 10. Although these measures quantify attention distribution and intensity, they should not be interpreted as pixel-level lesion overlap metrics.

**Table 10:** Class-wise quantitative Grad-CAM activation analysis of the proposed ConvNeXt-SAMC model.

Disease class	Correct samples	Activation coverage (%)	Mean CAM intensity
AMD	350	13.46 ± 3.91	0.1918 ± 0.0275
CNV	338	6.44 ± 1.85	0.0874 ± 0.0187
CSR	350	12.61 ± 3.68	0.1702 ± 0.0278
DME	335	6.41 ± 2.42	0.0900 ± 0.0258
DR	350	10.67 ± 3.44	0.1647 ± 0.0260
DRUSEN	336	6.30 ± 2.08	0.0837 ± 0.0197
MH	350	9.98 ± 2.67	0.1447 ± 0.0200
NORMAL	346	5.91 ± 2.31	0.0967 ± 0.0226

#### 4.6.3 LIME explanation

To further improve the interpretability of the proposed ConvNeXt-SAMC framework, Local Interpretable Model-Agnostic Explanations (LIME) were employed. LIME identifies the image regions that contribute most to the model's predictions. Unlike gradient based methods, it generates explanations through input perturbations. The method approximates the model locally using an interpretable surrogate model. As a result, LIME provides complementary insights into the model's decision-making behavior.

In this study, LIME explanations were obtained using superpixel segmentation with controlled compactness. Additional steps such as morphological filtering, connected component analysis and region selection were used to remove noisy activations. The resulting regions seem to be more spatially consistent and correspond to clinically relevant areas.

**Figure 9:**
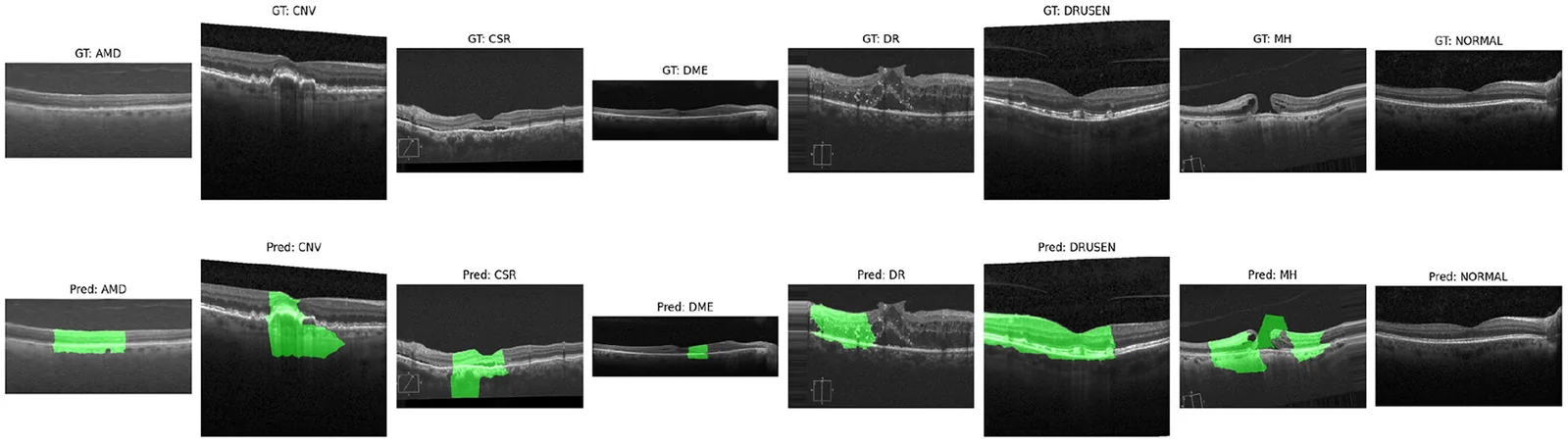
Representative LIME explanations for OCT images of each of the eight retinal disease classes The **top** row presents the original images, while the **bottom** row shows the corresponding LIME visualizations indicating the regions that influenced the classification decisions.

Representative samples were randomly chosen for LIME analysis. Figure 9 illustrates that the framework proposed here is concerned with the most important areas of the retina from a diagnostic perspective, irrespective of the disease type. For example, the target regions in CNV and DME diseases are identified by the presence of fluid accumulation below and inside the retina, respectively. In contrast, for DRUSEN and MH types, the identified regions correspond to structural changes such as drusen formation and macular hole presence. Notably, there is negligible activation for NORMAL images, indicating that the framework does not rely on any misleading information.

The majority of the explanations are well localized, but there are some difficult cases with slightly wider activation regions. This reflects the intrinsic ambiguity of interpretation of OCT images and the complexity of retinal disease morphology. However, the highlighted regions are still largely consistent with clinically relevant pathological structures. This consistency shows that the proposed model can predict with high accuracy and make decisions that are reliable and interpretable.

### 4.7 Ablation study

To evaluate the contribution of the individual components and design choices in the proposed ConvNeXt-SAMC framework, ablation study was conducted. The experiments study the role of the SAMC module, the effect of different dilation configurations, and the influence of temperature scaling and Monte Carlo (MC) Dropout equivalent results. Table 11 presents the corresponding results.

**Table 11:** Ablation results for the ConvNeXt-SAMC framework.

Model variant	Acc (%)	Precision	Recall	F1	ECE ↓
Baseline ConvNeXt	97.79	0.9781	0.9779	0.9778	0.0795
ConvNeXt + SAMC (1)	98.18	0.9819	0.9818	0.9818	0.0794
ConvNeXt + SAMC (1, 2)	97.89	0.9790	0.9789	0.9789	0.0790
ConvNeXt + SAMC (1, 2, 3)	**98.39**	**0.9840**	**0.9839**	**0.9839**	0.0785
ConvNeXt + SAMC + Temperature scaling	98.39	0.9840	0.9839	0.9839	0.0386
ConvNeXt + SAMC + Temperature scaling + MC dropout	98.39	0.9840	0.9839	0.9839	0.0848

Results are reported after incorporating SAMC, temperature scaling, and MC Dropout. Temperature scaling reduces the Expected Calibration Error (ECE) while maintaining classification performance. Bold values indicate the best-performing results obtained among the evaluated ablation configurations.

The baseline ConvNeXt-Tiny model achieved an accuracy of 97.79% with an F1-score of 0.9778. Incorporating the SAMC module improved the classification performance to 98.39% accuracy and an F1-score of 0.9839. This improvement demonstrates the effectiveness of adaptive multi-scale contextual feature extraction in enhancing discriminative feature representation for retinal OCT classification. The improvement between the baseline ConvNeXt-Tiny and the proposed ConvNeXt-SAMC model was further evaluated using the McNemar test, which confirmed that the observed difference in classification performance was statistically significant.

To further investigate the influence of multi-scale contextual aggregation, additional hyperparameter ablation experiments were performed by varying the dilation configuration of the SAMC module. Three configurations were evaluated: a single-scale configuration with a dilation rate (1), a two-scale configuration with dilation rates (1, 2), and the proposed three-scale configuration with dilation rates (1, 2, 3). The single-scale configuration achieved an accuracy of 98.18% and an F1-score of 0.9818, whereas the two-scale configuration achieved an accuracy of 97.89% and an F1-score of 0.9789. The proposed three-scale configuration achieved the best overall performance among the evaluated SAMC configurations, with an accuracy of 98.39% and an F1-score of 0.9839. These findings indicate that the effectiveness of SAMC depends on the complementary information provided by receptive fields of different spatial extents. Results further indicate that an increase in the number of dilation branches does not necessarily lead to a monotonic improvement. Nonetheless, the full three-scale setup provides the best combination among the evaluated configurations.

The influence of temperature scaling was evaluated separately, as its objective differs from that of the SAMC module. SAMC modifies the feature representation and enhances the classification performance. Temperature scaling is a *post-hoc* calibration method that adjusts the confidence of the predicted probabilities while keeping the predicted class labels unchanged. As shown in Table 11, temperature scaling reduced the ECE from 0.0785 to 0.0386 while maintaining an accuracy of 98.39%. Therefore, the unchanged classification accuracy after temperature scaling is expected, as its contribution lies in improving the reliability of the predicted probabilities rather than diagnostic discrimination.

To statistically validate the observed calibration improvement, a paired bootstrap analysis with 1000 resampling iterations was performed using the uncalibrated and temperature-scaled predictions obtained from the same test samples. The analysis produced a mean bootstrap ECE reduction of 0.03983, with a 95% confidence interval of [0.03472, 0.04481]. The confidence interval excluded zero, and the two-sided bootstrap analysis indicated a statistically significant reduction in calibration error (*p* < 0.001). These results demonstrate that the reduction in ECE obtained through temperature scaling is statistically reliable and is unlikely to be explained by sampling variation alone.

MC Dropout was incorporated for predictive uncertainty estimation rather than classification performance enhancement. Accordingly, the unchanged classification metrics after the application of MC Dropout are consistent with its intended purpose. Multiple stochastic forward passes provide information regarding prediction variability and model uncertainty, which can complement deterministic class predictions in clinical decision-support applications. Therefore, the contribution of MC Dropout should be interpreted in terms of uncertainty characterization rather than improvement in conventional diagnostic accuracy metrics.

Taken together, the ablation results demonstrate that the components of the proposed framework serve distinct and complementary functions. The SAMC module improves discriminative feature representation through adaptive multi-scale contextual modeling, temperature scaling significantly improves the calibration of predicted probabilities without altering diagnostic decisions, and MC Dropout provides additional information regarding predictive uncertainty. The additional dilation configuration experiments and statistical analysis of ECE variation provide further evidence supporting the architectural design and reliability assessment of the proposed ConvNeXt-SAMC framework.

### 4.8 Summary of performance gains

The ConvNeXt-SAMC model improves over the baseline in accuracy and macro F1 score. The results indicate better separation between classes.

The ECE becomes low after the application of the temperature scaling method. The predictions become close to the actual observations. The results of experiments on noise, blur, and brightness changes showed small changes, which mean that the model is stable.

McNemar test showed a *p*-value < 0.05. This means that there is a statistical difference when compared to the baseline model. This result suggests that the observed improvement is unlikely to be caused by random variation.

The results demonstrate the effectiveness of multi-scale feature extraction. The model shows the ability to recognize both simple and complex retinal patterns. Therefore, it is able to sustain consistent performance regardless of varying pathological conditions.

### 4.9 Comparison with existing feature enhancement modules

To further investigate the effectiveness of the proposed Spatial Adaptive Multi-Scale Convolution (SAMC) module, we performed additional experiments by incorporating three commonly used feature enhancement strategies, namely, Atrous Spatial Pyramid Pooling (ASPP), Convolutional Block Attention Module (CBAM), and Feature Pyramid Network (FPN) into the ConvNeXt-Tiny backbone.

All comparison models were trained and evaluated on the same OCT-C8 dataset splits, preprocessing pipeline, augmentation strategy, optimization settings, and evaluation protocol. This ensures a fair comparison between the proposed SAMC module with current feature enhancement methods.

The performance comparison of different ConvNeXt-Tiny variants is shown in Table 12. The results show that the proposed ConvNeXt-SAMC framework achieves comparable classification performance with ASPP, CBAM and FPN based models. The proposed model achieves 98.39% accuracy, 98.39 macro F1 score. The comparison not only emphasizes classification performance but also the computational characteristics of each feature refinement technique, reporting the number of parameters, FLOPs, inference time, throughput, and memory consumption.

**Table 12:** Performance and computational efficiency comparison of ConvNeXt-Tiny with different feature refinement modules.

Model	Acc. (%)	F1	Recall	Precision	Params	FLOPs	Time	FPS	Memory
ConvNeXt + CBAM	98.00	0.9800	0.9800	0.9801	27.93	4.46	7.910	126.42	0.888
ConvNeXt + ASPP	98.18	0.9818	0.9818	0.9819	51.34	7.93	13.746	72.75	1.521
ConvNeXt + FPN	98.29	0.9828	0.9829	0.9829	35.67	5.61	47.758	20.94	0.734
**ConvNeXt-SAMC (Proposed)**	**98.39**	**0.9839**	**0.9839**	**0.9840**	**30.22**	**4.82**	**13.645**	**73.29**	**0.484**

Bold values indicate the best-performing results among the compared feature refinement modules.

SAMC achieves comparable performance as it performs stage-wise multi-scale spatial refinement in the ConvNeXt hierarchy. Unlike ASPP that uses parallel atrous convolutions for contextual aggregation and FPN that mainly focuses on hierarchical feature fusion, SAMC progressively refines spatial representations after each stage of the backbone. This allows for efficient modeling of retinal abnormalities at different spatial scales, while maintaining the computational efficiency of the ConvNeXt architecture.

### 4.10 Comparative analysis with state of the art methods

Table 13 presents the comparison of the ConvNeXt-SAMC model against recent methods for retinal OCT image classification. The comparison covers the models based on CNNs as well as the hybrid architectures. This comparison is helpful in thoroughly assessing the proposed model against the present state-of-the-art methods.

**Table 13:** Comparison of the proposed ConvNeXt-SAMC model with existing retinal OCT classification methods.

Model	Accuracy	F1	Recall	Precision	Spec	Calibration assessment	Robustness evaluation
Swin-poly transformer (He et al., 2023)	97.12	97.10	97.13	97.13	—	×	×
HTC-retina (Laouarem et al., 2024)	97.00	97.01	97.00	97.04	99.56	×	×
ResNet34 (modified residual) (Karthik and Mahadevappa, 2023)	92.4	92.0	92.0	93.0	—	×	×
ResNet50 (modified residual) (Karthik and Mahadevappa, 2023)	90.3	91.0	91.0	91.0	—	×	×
ResNet101 (modified residual) (Karthik and Mahadevappa, 2023)	86.1	86.0	86.0	86.0	—	×	×
Lightweight CNN + Transf. (Pan et al., 2025)	98.5	97.99	97.99	98.02	99.71	×	×
WaveNet-SF (Cheng et al., 2025)	97.82	97.82	97.82	97.82	—	×	×
MSLI-Net (Qi et al., 2025)	96.72	96.72	96.72	96.75	—	×	×
**Proposed ConvNeXt–SAMC**	**98.39**	**98.39**	**98.39**	**98.40**	**99.77**	**✓**	**✓**

The results show that the proposed approach achieves competitive performance across accuracy, F1-score, recall, and precision, while also maintaining higher specificity. Unlike several earlier methods, it additionally provides calibration and robustness characteristics. Bold values indicate the best-performing results among the compared retinal OCT classification methods.

The proposed model demonstrates 98.39% accuracy. It also displays high performance in terms of F1 score, recall, and precision. Transformer-based models such as Swin Poly and hybrid architectures such as HTC Retina, demonstrate accuracies of approximately 97%. Although some recent CNN-Transformer models achieve slightly higher performance in certain cases, the proposed model remains competitive while maintaining a relatively efficient architecture. Recent multi-scale approaches, including WaveNet-SF and MSLI-Net, are also included in the comparison. WaveNet-SF reports an accuracy of 97.82%, with an F1-score, recall, and precision of 97.82%, 97.82%, and 97.83%, respectively, whereas MSLI-Net reports 96.72% accuracy, 96.72% F1-score, 96.72% recall, and 96.75% precision. In comparison, the proposed ConvNeXt-SAMC achieves 98.39% accuracy, F1-score, and recall, together with 98.40% precision, demonstrating competitive and balanced performance across the reported classification metrics.

Most existing works focus on classification performance. Fewer studies report calibration or robustness. As seen in Table 13, these aspects are often not included, even though they are important in clinical use.

The ConvNeXt-SAMC model includes both calibration and robustness evaluation. Calibration compares predicted probabilities with observed outcomes. Robustness checks how the model responds to variations in the input.

Overall, while certain recent approaches report marginally higher or comparable accuracy in specific cases, the proposed ConvNeXt-SAMC model demonstrates a more balanced performance across all evaluation metrics, including F1-score, precision, recall, and specificity. In addition to competitive classification accuracy, the model consistently maintains higher calibration quality and improved robustness compared to existing methods, which are often not jointly reported in prior studies. This indicates that the proposed framework does not only focus on classification accuracy but also ensures more reliable and well-calibrated predictions, which are critical for clinical deployment in real-world retinal screening scenarios.

### 4.11 Computational complexity and efficiency analysis

The computational efficiency of the proposed ConvNeXt-SAMC model was evaluated in terms of parameter count, FLOPs, inference time, throughput, and peak GPU memory usage. The model contains 30.22 million parameters and requires 4.82 GFLOPs for a single forward pass at an input resolution of 224 × 224. The average inference time is 13.645 ms per image, corresponding to 73.29 FPS, with a peak GPU memory usage of 0.484 GB.

A comparison with recent OCT classification methods, including WaveNet-SF and MSLI-Net, is presented in Table 14. Since these methods do not consistently report all computational metrics, only the values available in their original studies are included.

**Table 14:** Computational complexity and efficiency comparison.

Method	Params(M)	FLOPs(G)	Inference time (ms)
WaveNet-SF (Cheng et al., 2025)	27.84	–	511.8
MSLI-Net (Qi et al., 2025)	89.69	33.46	–
Proposed ConvNeXt–SAMC	30.20	4.82	13.645

To further examine the performance–efficiency trade-off under a controlled setting, the proposed SAMC module was compared with CBAM, ASPP, and FPN variants implemented using the same ConvNeXt-Tiny backbone and evaluated under identical experimental conditions. The results are summarized in Table 12.

The results show that SAMC provides a favorable balance between classification performance and computational efficiency. CBAM achieves the lowest parameter count and highest throughput, whereas ASPP introduces substantially higher parameter and memory requirements. FPN achieves moderate computational complexity but considerably higher inference latency. In comparison, SAMC maintains the highest classification performance in the comparison while requiring only 4.82 GFLOPs and the lowest peak GPU memory usage of 0.484 GB.

### 4.12 Cross dataset generalization

To study how well the proposed ConvNeXt-SAMC model generalizes, a cross dataset experiment was performed. The model was trained on the OCT-C8 dataset and then tested on the independent Kermany OCT2017 Version 2 dataset test split (Mooney, 2018) obtained from the Kaggle repository. The external evaluation set consisted of 968 images, with 242 images per class (CNV, DME, DRUSEN, and NORMAL). No image filtering or exclusion was performed during evaluation. Since the Kermany dataset contains only four classes (DRUSEN, NORMAL, CNV, and DME), these were mapped to their corresponding labels in the OCT-C8 setup. During evaluation, predictions were restricted to the logits of the mapped classes. This way, the comparison could be carried out directly without retraining the model.

No fine-tuning or additional training was performed on the Kermany dataset. The model was evaluated directly on the dataset without any further adaptation. Therefore, the results reflect the model's performance under domain shift.

#### 4.12.1 Performance on external dataset

**Figure 10:**
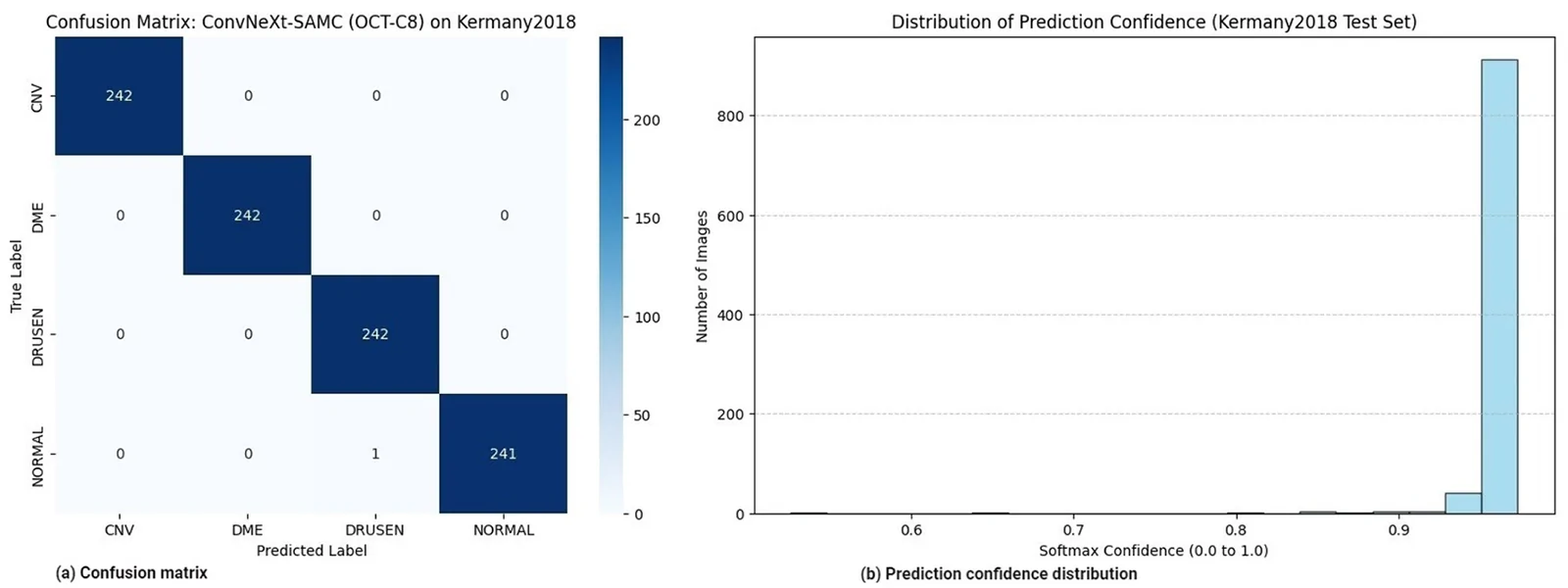
External validation of the proposed ConvNeXt-SAMC model on the Kermany OCT2017 Version 2 test split (968 images). **(a)** Confusion matrix showing class-wise prediction outcomes, with only one misclassification observed between the NORMAL and DRUSEN classes. **(b)** Distribution of prediction confidence scores, indicating that the majority of predictions are assigned high confidence, thereby demonstrating strong generalization on the external dataset.

The reported external accuracy was obtained using all 968 images from the official Kermany OCT2017 Version 2 test split. The model achieved an overall accuracy of 99.90% on Kermany OCT2017 Version 2 dataset. Class-wise precision, recall, and F1-scores are summarized in Table 15. The confusion matrix shown in Figure 10a reveals only a single misclassification. This occurred between the NORMAL and DRUSEN classes. The error may be attributed to similarities in their OCT structural patterns.

**Table 15:** Performance evaluation of the ConvNeXt-SAMC framework on the Kermany OCT2017 Version 2 external test split (968 images).

Class	F1-score	Recall	Precision
NORMAL	0.99	1.00	0.99
DRUSEN	1.00	1.00	1.00
CNV	1.00	1.00	1.00
DME	1.00	1.00	1.00
**Total accuracy**	**99.90%**		

Bold value indicates the overall classification accuracy obtained on the Kermany OCT2017 Version 2 external test set.

The results show that the learned representations are transferable for the model. The model effectively handled differences in acquisition settings and data distributions across the tested datasets.

#### 4.12.2 Data leakage analysis

To verify the integrity of the cross-dataset evaluation, a hash-based duplicate detection procedure was performed using MD5 hashing (Rivest, 1992). Training images were compared against Kermany OCT2017 Version 2 test set images. No duplicate samples were found. Therefore, the leakage rate was 0.00%. This finding shows that the reported performance was not influenced by any overlap between the training and test datasets.

#### 4.12.3 Confidence analysis

Figure 10b shows the distribution of prediction confidence scores. The average confidence score was 0.9599. Most predictions were concentrated at higher confidence levels.

Only a small number of samples exhibited lower confidence values. The minimum confidence score is 0.5255. These cases generally corresponded to more challenging predictions. This suggest that the model reduces its confidence under uncertainty rather than producing overconfident outputs.

#### 4.12.4 Interpretability on external dataset

**Figure 11:**
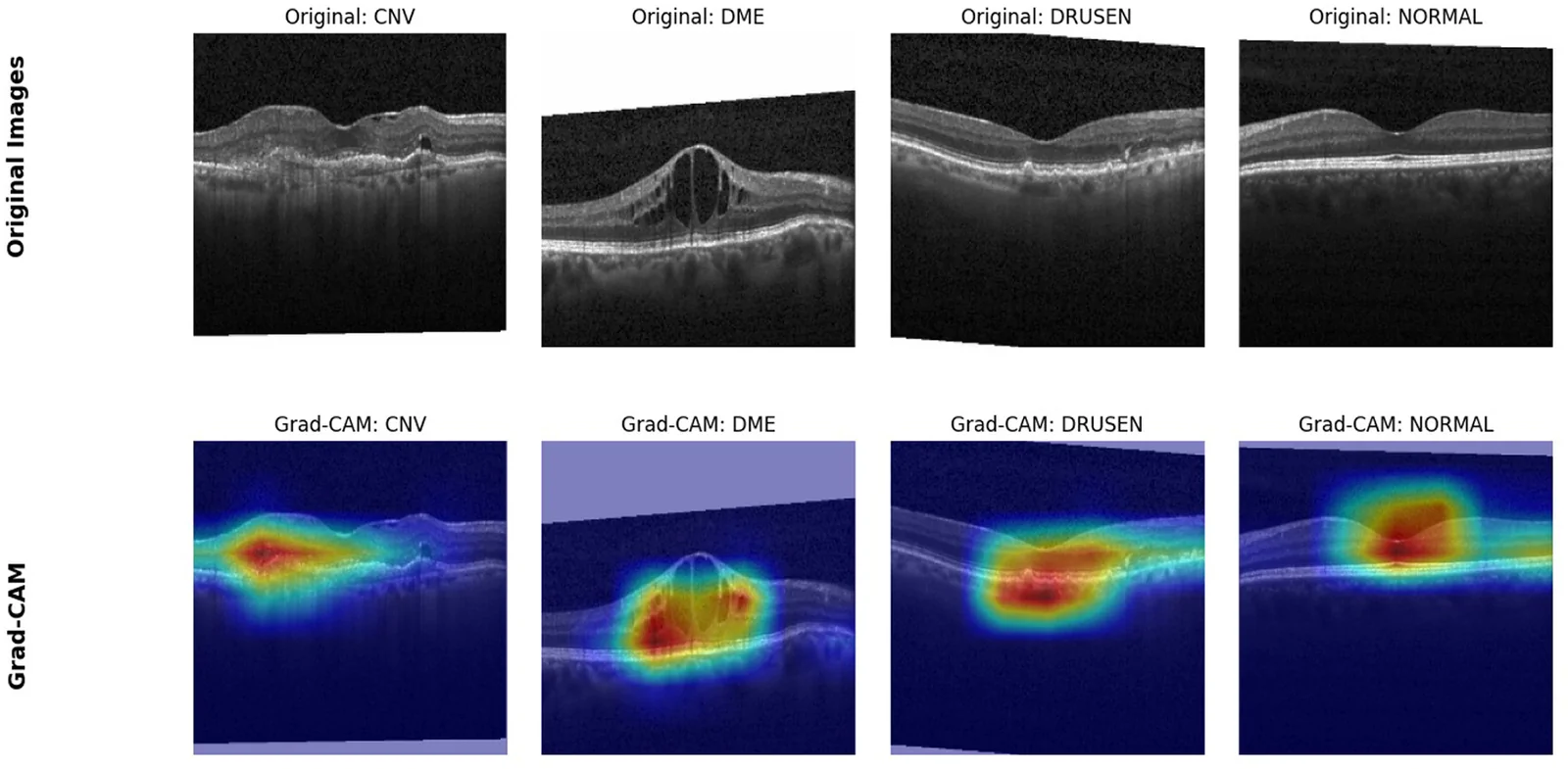
Grad CAM visualizations of the proposed ConvNeXt–SAMC model on the Kermany OCT2017 Version 2 dataset.

Grad-CAM visualizations were generated using representative samples from the Kermany OCT2017 Version 2 dataset. The attention maps consistently highlight retinal regions relevant to each class. Figure 11 illustrates Grad-CAM visualizations obtained from representative samples in the Kermany OCT2017 Version 2 dataset. The activations are concentrated in areas with fluid accumulation and structural abnormalities for CNV and DME. In DRUSEN samples, the model focuses on regions containing sub-retinal deposits. In contrast, NORMAL samples show a broader distribution of attention across the retinal layers.

These results indicate that the proposed ConvNeXt–SAMC framework learns feature representations that generalize across datasets. The model also maintains consistent and clinically meaningful interpretability.

## 5 Discussion

The study presents an approach for enhancing the classification of retinal OCT images using the SAMC block as part of the ConvNeXt architecture. The new design shows an advantage in the aspect of classification quality when compared with the baseline architecture while maintaining efficiency in computations. The better result in the proposed architecture is achieved because of the multiscale representation of the data by SAMC. Unlike regular convolutional layers that have predefined receptive fields, parallel dilated depthwise convolutions and adaptive fusion mechanism capture both fine retinal textures and larger anatomical structures. This allows feature representations to be refined progressively across network stages.

The proposed approach demonstrated consistent performance for all the eight retinal types. Perfect classification was achieved for AMD, CSR, DR, and MH classes. This indicates clear discriminative properties within the learned feature space. Most of the misclassifiations took place between CNV, DME, DRUSEN, and NORMAL classes. In these classes, morphological overlapping leads to difficulties in classifying them. Despite these challenges, the model proved to be robust enough to deal with retinal pathologies with different structures.

In addition to classification performance, probability calibration analysis was performed to evaluate the confidence estimates of the model predictions. Temperature scaling reduced the discrepancy between predicted confidence and observed outcomes without altering the classification performance. The statistical analysis of ECE variation further confirmed that the observed calibration improvement was significant, demonstrating that temperature scaling improves the reliability of predicted probabilities rather than diagnostic discrimination. Monte Carlo Dropout provided estimates of predictive variability, with higher uncertainty observed for more challenging samples. The ablation analysis therefore demonstrates that the evaluated components serve distinct and complementary functions: SAMC primarily improves discriminative feature representation and classification performance, temperature scaling improves probability calibration without changing class predictions, and MC Dropout provides additional information regarding predictive uncertainty through stochastic inference. Together, these evaluations provide a more comprehensive assessment of model behavior beyond classification accuracy and may support further analysis of model reliability in clinical decision-support settings.

The effectiveness of the network in coping with different distortions such as Gaussian noises, blurring, and variations in brightness was tested. Very little degradation in performance was reported. This shows that the model is robust in the face of possible disturbances that may occur during the process of acquiring images using OCT technology. The observed robustness is related to the progressive multi-scale contextual modeling introduced by SAMC. The module extracts features from complementary receptive fields. This helps the model to handle variations in image quality and acquisition conditions more effectively.

External validation was done using the Kermany OCT2017 Version 2 dataset. Training for the model was only done on the OCT-C8 data. No more training and fine-tuning were conducted prior to evaluation on the external dataset. Strong performance was achieved by the model on the external dataset. This indicates good feature transfer capability of the learned features on datasets acquired under varying conditions. Duplicates analysis did not find any overlapping data in the OCT-C8 and Kermany datasets. Grad-CAM analysis showed that the model paid attention to clinically meaningful areas of the retina during predictions.

Several retinal OCT classification approaches, including transformer-based architectures (Dosovitskiy et al., 2021; Liu et al., 2021; He et al., 2023), hybrid CNN-transformer models (Laouarem et al., 2024), and lightweight attention-based frameworks (Pan et al., 2025), report strong classification performance. Recent multi-scale approaches have also explored alternative mechanisms for capturing complementary retinal features. WaveNet-SF (Cheng et al., 2025) combines wavelet-based representation with multi-scale spatial feature learning, while MSLI-Net (Qi et al., 2025) employs multi-scale and multi-level feature integration for retinal OCT classification. These approaches further demonstrate the importance of multi-scale representation learning for distinguishing retinal pathologies with variations in lesion size and structural appearance. However, while much of the current literature focuses mainly on classification accuracy, the current study takes a further step to investigate the following aspects: calibration, uncertainty estimation, robustness, statistical significance, interpretability, and generalization across datasets. These aspects provide further insight into the model's performance beyond accuracy.

There are some limitations for the work. The cross dataset validation was performed but only on a small number of test data. Multi-center large datasets also need to be explored. These datasets could comprise images collected from various OCT devices. This would offer more conclusive evidence for generalizability of the model. Also, the present system is only for image-level classification; and does not include lesion localization or segmentation. Future work can look into domain adaptation, segmentation assisted learning and optimization for real time clinical adaptation.

In summary, the newly introduced framework of ConvNeXt–SAMC enhances the performance of retinal OCT classification through adaptive multi-scale contextual modeling, which does not affect computation speed negatively. This model performs well under multiple evaluation criteria such as calibration, robustness, interpretability, and generalization. Collectively, these demonstrate that the model could be utilized in clinical decision-making systems.

## 6 Conclusion

In this study, a ConvNeXt-Tiny architecture enhanced with the Spatial Adaptive Multi-Scale Convolution (SAMC) module was proposed for multi-class retinal OCT classification. The architecture employs parallel depth-wise convolutions with different dilation rates along with adaptive fusion to account for the various retinal features at multiple scales. Hence, consistent classification accuracy across diverse disease classes was obtained. Experimental analysis on the OCT C8 dataset demonstrates high classification accuracy alongside a comprehensive assessment of the proposed model's reliability. Temperature scaling improved probability calibration, whereas Monte Carlo Dropout provided uncertainty estimates based on stochastic predictions. The network is also capable of handling noise, blur, and illumination perturbations robustly. Statistical significance of the achieved performance gain is verified through McNemar's test. Cross-dataset analysis on the Kermany 2018 dataset, without any further learning, achieved 99.90% classification accuracy. This indicates generalization under distribution shift. According to Grad-CAM and LIME analyses, the proposed model focuses on clinically significant retinal regions for making predictions. The results clearly demonstrate that the ConvNeXt-SAMC architecture can classify retinal OCT accurately and reliably. The model achieves strong classification performance, while calibration, uncertainty, robustness, and interpretability analyses provide additional characterization of its prediction behavior. Future work will focus on large scale multicenter validation, domain adaptation, and multi modal retinal imaging to further improve generalization and clinical applicability.

## Data Availability

The original contributions presented in the study are included in the article/supplementary material, further inquiries can be directed to the corresponding author.
